# Combined administration of mesenchymal stem cells overexpressing IGF-1 and HGF enhances neovascularization but moderately improves cardiac regeneration in a porcine model

**DOI:** 10.1186/s13287-016-0350-z

**Published:** 2016-07-16

**Authors:** Guadalupe Gómez-Mauricio, Isabel Moscoso, María-Fernanda Martín-Cancho, Verónica Crisóstomo, Cristina Prat-Vidal, Claudia Báez-Díaz, Francisco M. Sánchez-Margallo, Antonio Bernad

**Affiliations:** Jesús Usón Minimally Invasive Surgery Center, Cáceres, Spain; Department of Cardiovascular Development and Repair, Fundación Centro Nacional de Investigaciones Cardiovasculares Carlos III, Madrid, Spain; Cardiovascular Area, CIMUS, Instituto de Investigación Sanitaria, University of Santiago de Compostela, Santiago de Compostela, Spain; ICREC (Heart Failure and Cardiac Regeneration) Research Program, Health Sciences Research Institute Germans Trias i Pujol (IGTP), Badalona, Barcelona, Spain; Department of Immunology and Oncology, Centro Nacional de Biotecnología (CNB-CSIC), Darwin 3 (Campus UAM Cantoblanco), 28049 Madrid, Spain

**Keywords:** Mesenchymal stem cells, HGF, IGF-1, Acute myocardial infarction, Porcine model, Gene therapy, Cell therapy

## Abstract

**Background:**

Insulin-like growth factor 1 (IGF-1) and hepatocyte growth factor (HGF) are among the most promising growth factors for promoting cardiorepair. Here, we evaluated the combination of cell- and gene-based therapy using mesenchymal stem cells (MSC) genetically modified to overexpress IGF-1 or HGF to treat acute myocardial infarction (AMI) in a porcine model.

**Methods:**

Pig MSC from adipose tissue (paMSC) were genetically modified for evaluation of different therapeutic strategies to improve AMI treatment. Three groups of infarcted Large White pigs were compared (I, control, non-transplanted; II, transplanted with paMSC-GFP (green fluorescent protein); III, transplanted with paMSC-IGF-1/HGF). Cardiac function was evaluated non-invasively using magnetic resonance imaging (MRI) for 1 month. After euthanasia and sampling of the animal, infarcted areas were studied by histology and immunohistochemistry.

**Results:**

Intramyocardial transplant in a porcine infarct model demonstrated the safety of paMSC in short-term treatments. Treatment with paMSC-IGF-1/HGF (1:1) compared with the other groups showed a clear reduction in inflammation in some sections analyzed and promoted angiogenic processes in ischemic tissue. Although cardiac function parameters were not significantly improved, cell retention and IGF-1 overexpression was confirmed within the myocardium.

**Conclusions:**

The simultaneous administration of IGF-1- and HGF-overexpressing paMSC appears not to promote a synergistic effect or effective repair. The combined enhancement of neovascularization and fibrosis in paMSC-IGF-1/HGF-treated animals nonetheless suggests that sustained exposure to high IGF-1 + HGF levels promotes beneficial as well as deleterious effects that do not improve overall cardiac regeneration.

**Electronic supplementary material:**

The online version of this article (doi:10.1186/s13287-016-0350-z) contains supplementary material, which is available to authorized users.

## Background

Despite the effectiveness of current treatments, which have greatly reduced short-term morbidity and mortality of acute myocardial infarcted patients [1, 2], they are unable to prevent cardiac degeneration due to massive tissue damage. The gold standard technique is heart transplant, a complex surgery with serious limitations, including donor shortage and possible rejection, which prompts the search for therapeutic alternatives.

Cell therapy and regenerative medicine have raised great expectations in recent years [3]. Initially, an autologous myoblast grafting trial concluded that they did not integrate into the host myocardium or transdifferentiate into cardiomyocytes, despite confirmation of some beneficial effects on myocardial function [4]. More recent studies suggest that skeletal muscle-derived stem cells (naive, preconditioned, or genetically modified) have the ability to adopt a cardiomyocyte phenotype in vitro and in vivo [4, 5]. Stem cells are now being assessed as a therapeutic tool in the treatment of ischemic heart disease, with some promising initial results. These studies attempt to minimize the evolution of damaged tissue after acute myocardial infarction (AMI) and promote repair of affected cardiac structures [6, 7]. In murine models, adult stem cell transplant in the heart leads to improved cardiac function and neoangiogenesis [8, 9]. Cell therapy is thus an alternative strategy whose main objective is to repair damage and/or delay its evolution after ischemic stroke.

These promising initial findings led to the development of numerous clinical trials of cardiac cell therapy. Results have confirmed the safety of these procedures, especially when autologous cells are used. Currently, more than 850 clinical trials for cardiovascular disease have been conducted, and more than 60 are specific for AMI treatment [10]. Whereas most use mesenchymal stem cells (MSC) from bone marrow (BM-MSC), some use MSC obtained from adipose tissue or from umbilical cord, and even cardiac stem/progenitor cells (CSC/CPC). For example, the phase I completed APOLLO clinical trial used adipose tissue-derived stem cells [11] and the TECAM2 clinical trial used hematopoietic stem cells to treat AMI [12]. Administration routes differed in each of these; in the latter, treatment was administered by intracoronary injection, whereas APOLLO used a device called NOGA™ (cardiac mapping system) to allow transendocardial cell administration.

The concept of the heart as a post-mitotic organ incapable of self-renewal has changed in recent years. Early studies showed that some cardiomyocytes can re-enter the cell cycle [13, 14] and lead to limited regeneration [13, 14]. A possible stem cell population was soon identified in the adult rodent heart, whose regeneration potential was analyzed in animal models of AMI [15]. In many studies, this population of CSC/CPC is the main objective as an alternative to other stem cell types used to date. Only three clinical trials have used CSC (SCIPIO, CADUCEUS, and TICAP), with promising results [16–18]. Based on very promising preclinical results, an additional phase I/IIa clinical trial (CARE-MI) was recently launched, in which 55 patients are being treated with allogeneic CSC/CPC (EudraCT 2013-001358-81).

The therapeutic capacity of CSC/CPC is attributed mainly to their potential to differentiate into many distinct reparative cell types. There is nonetheless insufficient documentation of efficient differentiation of transplanted CPC into therapeutically relevant numbers of functional reparative cells in injured tissues [19]. Low survival appears to be one of the main factors, although limited availability of functional niches and deficient early vascularization could also be responsible. A substantial part of these repair effects might not be mediated mainly by direct differentiation of engrafted CPC, but rather by CPC-secreted paracrine factors [20]. These factors are postulated to promote survival and arteriogenesis [21], protect against myocardial ischemia, and stimulate endogenous repair and regeneration pathways, resulting in durable benefits despite evanescent survival of transplanted cells [22].

Several reports suggest that various stem cell-secreted substances, such as growth factors, mediate angiogenesis and protect against myocardial ischemia. Some studies showed the angiogenic and anti-apoptotic properties of hepatocyte growth factor (HGF) [23], and insulin-like growth factor-1 (IGF-1) has cardioprotective properties and beneficial effects on the heart [24]; this role in myocardial repair has led to their therapeutic evaluation. IGF-1 and HGF are a good option to be considered for such therapies, and their direct administration has been evaluated for potential AMI treatment in porcine models [25].

Another strategy in treatment for cardiac regeneration combines cell and gene therapy to enhance their efficacy. Many studies have been performed to improve the effectiveness of cardiovascular therapy using a variety of populations, including CSC and cardiomyocyte progenitors genetically engineered using the αMHC promoter [26]. Although the effects of administering different cell types or growth factors, separately and independently, have been analyzed [27, 28], few studies have examined their combined effect [29]. Here, we explored the combination of cell and gene therapy strategies using MSC genetically modified to overexpress IGF-1 or HGF growth factors as a single treatment in a porcine AMI model.

## Methods

### Cell culture and gene modifications on paMSC

The isolation of pig MSC from adipose tissue (paMSC) from lipoaspirates of pigs was carried out according to previous MSC isolation protocols in humans [30, 31] and pigs [32, 33]. Briefly, ventral adipose tissue was washed twice with phosphate-buffered saline (PBS) and was subjected to enzymatic digestion at 37 °C for 1 h with collagenase V (Sigma; 0.075 % final concentration). Digested tissue was centrifuged at 450 g for 10 min, and the cell suspension was filtered through two layers of nylon chiffon (70 μm mesh). paMSC were plated (5 × 10^3^ cells/cm^2^) and maintained in Dulbecco’s modified Eagle’s medium (DMEM) supplemented with 10 % fetal bovine serum (FBS), 1 % l-glutamine and 1 % penicillin/streptomycin solution (37 °C, 5 % CO_2_). After 7 days, and when cultures had reached a confluence of approximately 80 % (passage 0), cells were trypsinized (0.05 % Trypsin–EDTA; Invitrogen) and seeded at a density of 5 × 10^3^ cells/cm^2^ (passage 1). In some experiments, growth was compared in distinct O_2_ conditions (20 % vs 3 % O_2_).

paMSC-IGF-1-green fluorescent protein (GFP)- and paMSC-HGF-Cherry were generated by stable transduction with lentiviral vectors encoding IGF-1-GFP (pRRLsin18.CMV-IGF-1-IRES-GFP) or HGF-Cherry (pRRLsin18.CMV-HGF-IRES-Cherry) sequences.

### Cell and molecular characterization of MSC

The International Society for Cellular Therapy (ISCT) has defined the minimal criteria to be met by cells to be considered MSC as the following [31]: adherence to plastic in standard culture conditions; positive phenotype: CD105, CD73, CD90 and negative phenotype: CD45, CD34, CD14 or CD11b (monocytes), CD79a or CD19 (lymphocytes); and in vitro differentiation to three mesoderm lineages: osteocytes, adipocytes and chondrocytes (demonstrated by staining of in vitro cell culture). All experiments were carried out in at least four cell isolations. The morphological evaluation of cells was performed by direct observation with a microscope (Nikon Eclipse TE-2000-S) and photographic record digital camera (Nikon DS-Fi1).

### Flow cytometry

Phenotypic analysis was performed by FACS (fluorescence-activated cell system). The antibodies used were CD90-FITC, CD105-FITC, CD44-FITC, CD29-FITC, CD31-FITC, and CD45-FITC. Mouse IgG1-FITC, mouse IgG2a-FITC, and rat IgG1-FITC were used as isotopic controls. After incubation, cells were washed with PBS (300 g, 5 min). Quantitative analyses were performed using a flow cytometer FACS Scan (BD Biosciences, CA, USA), as previously described [33].

The FACS technique was used to separate paMSC transduced with GFP which were not infected (group II). This technique was also used to separate the same cells transduced with pRRLsin.18.CMV-IGF-1-IRES-GFP or pRRLsin.18.CMV HGFIRES-Cherry vectors of negative cells in these transductions (group III; flow cytometer FACS Aria; BD Biosciences).

### paMSC in vitro differentiation

The pluripotency determination was carried out using cell differentiation assays into three lineages (adipocytes, chondrocytes, and osteocytes), as previously described [33]. We performed more than three independent sets of experiments.

Molecular studies of gene expression were performed with adipogenic differentiation markers (*PPAR-γ* and *LPL*) and osteogenic markers (*RUNX-2*, *BMP-2*, *BMP-6*, *GATA-4*, *COL1A1*, and *BGLAP*, which is equivalent to human osteocalcin) whose expression and/or activation is required for the commitment and progression of each particular differentiation pathway. In this analysis, RNA was extracted from all studied samples. As negative controls, cells grown in standard culture medium were used and, for test samples, the cells were grown in specific differentiation media for each test. These cDNA were amplified by reverse transcription polymerase chain reaction (RT-PCR) to examine differences in gene expression of markers involved in each differentiation [32].

### Karyotype and population doubling (PD) measure

After culture, 0.1 μg/ml colcemid was added for 4 h and metaphase cells were prepared by standard methods. Q-FISH was carried out using a FITC-labeled LL(CCCTAA)3 PNA telomeric probe (Eurogentec, Liège, Belgium) as described [34]. Cumulative PD was calculated with the formula PD = (log (Nn/Nn_1))/log 2 (n: passage; N: cell number).

### RT-qPCR

Total RNA was extracted from the different cell preparations (mature cells, induced, and control samples) using TRI REAGENT (Sigma-Aldrich) according to the manufacturer’s instructions. The total RNA concentration and purity was determinate using a Nano-Drop spectrophotometer (Thermo Scientific), and the 260/280 ratio generally ranged between 1.9 and 2.0 cDNA was synthesized from 1 μg of total RNA using random primers (Invitrogen) and SuperScript® III Reverse Transcriptase (Invitrogen) according to the manufacturer’s instructions. Primer sequences are listed in Additional file 1 (Table S1) and were designed using the web program of the National Center for Biotechnology Information (NCBI). The quantitative RT-PCR (RT-qPCR) was performed using SYBR Green (Applied Biosystems) in a Mastercycler® ep realplex (Eppendorf). RT-qPCR products were quantified using the 2^–ΔΔCt^ method, using GusB as ACTB as housekeeping genes (indicated in each analysis).

### Lentiviral vector transduction of paMSC

Lentiviral vectors were produced by transient calcium-phosphate transfection of 293T cells as previously described [35], and viral stocks with titers (transduction units; TU/mL) of 1–2 × 10^7^ TU/ml were prepared.

To generate paMSC-IGF and paMSC-HGF cells, paMSC were stably transduced (multiplicity of infection (MOI) = 50) with lentiviral vectors encoding IGF-1-GFP or HGF-Cherry. Lentivirus was added together with 8 μg/ml polybrene (Sigma, St Louis, MO, USA) in expansion medium for 12 h at 37 °C; fresh medium was added the next day, and culture maintained for 4–5 days. Transduced paMSC were sorted by GFP or Cherry fluorescence. Control transductions were conducted with empty vectors (pRRL-GFP or pRRL-Cherry).

### Immunocytochemistry

Immunohistochemistry was performed by standard methods. Primary and secondary antibodies used are listed in Additional file 2 (Table S2). Incubation with secondary antibody alone did not produce any detectable background signal. Slides were mounted in Hoechst 33342 (Invitrogen, Eugene, Oregon, USA), Vectashield with DAPI (Vector labs) or Prolong® Gold Antifade with DAPI (Invitrogen) and analyzed by confocal microscopy (Leica SP5, Leica). GFP+ cells in the myocardium were detected by immunohistochemistry with an anti-GFP antibody (see Additional file 2: Table S2) and a secondary antibody Alexa Fluor 568.

### In vivo studies

The study protocol was approved by the Jesús Usón Minimally Invasive Surgery Centre Animal Care and Use Committee and according to the European Agreement of Vertebrate Animal Protection for Experimental Use Guide (86/609). All procedures were performed as described [32], with indicated specific modifications.

Fourteen 3- to 4-month-old female Large White pigs (*Sus scrofa*) (39 ± 9.72 kg) were subjected to experimental AMI (distributed as one control animal without AMI and injected with superparamagnetic iron oxide (SPIO)-labeled GFP+ cells, an infarcted animal injected with the same (SPIO)-labeled cells and four animals with AMI in each of three treatment groups). Allogenic cell transplantation was performed the same day of the infarction by intramyocardial administration. A single dose (50 × 10^6^ viable cells/animal; *n* = 4 per group) was administered in a maximum volume of 1.5 ml saline. Thus, cell transplantation was performed using several injections (7–8 injections) in a volume of 0.2 ml of diluted cells (about 8 × 10^6^ cells per injection) distributed surrounding the infarction area. Group II received paMSC-GFP (50 × 10^6^ cells/animal), and group III received a combination of paMSC-IGF-1-GFP and paMSC-HGF-Cherry (each 25 × 10^6^ cells/animal); these animals were compared with controls infarcted but untreated with any cell type (control; group I). Pigs were evaluated by magnetic resonance imaging (MRI) before surgical intervention (T1, at baseline), and after 48 h (T2), with further studies at day 15 (T3) and day 30 (T4), prior to euthanasia and necropsy sampling. Left ventricular ejection fraction (LVEF), cardiac output (CO), stroke volume (SV), and infarcted area were measured. After 1 month, pigs were euthanized with an intravenous injection of potassium chloride solution (1–2 mmol/kg) to obtain samples for histological analysis.

### Analytical methods

To determine plasma concentrations of cardiac troponin I (cTnI), myocardial creatinine kinase (CKMB), and myoglobin levels, blood samples were collected at baseline, 48 h, and 15 and 30 days after AMI induction. After centrifugation, cardiac enzymes were measured by commercially available immunometric assays in a fluorometric immunoassay analyzer (AQT90 FLEX; Radiometer Medical ApS, Brønshøj, Denmark).

### MRI studies

Cardiac MRI was performed before the creation of the model (baseline), and at 48 h, 15 and 30 days after infarct induction and treatment using a 1.5 T MR system (Intera 1.5 T; Philips Medical Systems, Best, The Netherlands). All imaging was performed under general anesthesia using retrospective cardiac gating, with the animal in sternal decubitus and a four elements phase array coil placed around the animal’s chest. Images were acquired as previously described [32]. MR images were analyzed for left ventricular volume, mass, function, and infarct size.

### Histological analysis

Immediately after animal euthanasia, 1 month after AMI, samples were obtained from the infarction area (named as Heart 2, Heart 4, LV, and interventricular septum (IVS)) close to where the cell transplantation was performed and the hematoxylin/eosin (H/E) and Masson’s trichrome stainings were performed for the primary histological examination in 5-μm paraffin-embedded tissue sections. Inflammation, fibrosis, and new vessel formation were analyzed in H/E studies. The values presented correspond to the average obtained from each animal in each group (*n* = 4). The values assigned are: for inflammation, 0 when it was absent, 1 mild, 2 light, 3 moderate, and 4 severe; the presence of areas of fibrosis was set to 0 when the injury was not observed, 1 when its size was below 5 % of the sample, 2 between 5 and 30 %, 3 between 30 and 60 %, and 4 when greater than 60 %; neovascularization, value of 0 (none), 1 (few new vessels found), 2 (mild), 3 (moderate), and 4 (numerous new vessels).

### Immunohistochemistry

Pig heart paraffin-embedded sections were deparaffinized, rehydrated, washed with PBS, and antigens were retrieved with sodium citrate. For tyramide amplification, slides were incubated with 0.3 % H_2_O_2_. Tissue sections were then blocked and incubated overnight at 4 °C in blocking solution with primary antibody IGF-1 (Santa Cruz Biotechnology). Slides were washed and incubated for 1 h at room temperature with secondary antibodies conjugated to horseradish peroxidase (HRP) (see Additional file 2: Table S2). Incubation with secondary antibody alone did not give any detectable background signal. Vectastain ABC kit Elite PK-6101 (Vectorlabs) was used to develop DAB colorimetric assay.

### Western blot

paMSC cells were lysed in RIPA buffer containing protease inhibitors (Roche) and, after centrifugation, the supernatants were collected and protein quantified using the DC protein assay (Biorad). Protein (30 μg/lane) was loaded on 10–12 % SDS-polyacrylamide gels (Biorad) and Western blotting was performed. The membranes were blocked and incubated overnight with primary antibodies (see Additional file 2: Table S2) diluted in 5 % non-fat milk in PBS, 0.1 % Tween 20 (Sigma). Membranes were incubated with the corresponding HRP-conjugated secondary antibodies (see Additional file 2: Table S2) and developed with HRP SuperSignal® West Pico Trial (Thermo Scientific).

### Statistical analysis

Data are shown as mean ± SD. Non-parametric Kruskal-Wallis and Mann-Whitney tests were used to compare differences between groups for histological variables. Two-way analysis of variance (ANOVA) and Tukey’s tests were used in multiple comparisons for the remaining variables analyzed (cardiac enzymes and hemodynamic data). Values of *p* ≤ 0.05 were considered significant. All statistical computations were performed using SPSS 22.0 (IBM SPSS) and GraphPad Prism® 6.0.

## Results

### Growth characteristics and phenotypic and molecular characterization of primary paMSC

paMSC were isolated and expanded (passages 2–5) to obtain primary cultures (see Methods) and were characterized by flow cytometry. Immunophenotype analysis of the different paMSC subpopulations confirmed similarity with MSC from human and murine origins and matched ISCT criteria: cells were positive for CD90, CD105, CD29, and CD44, and negative for CD45 and CD31 (data not shown), as previously described [31].

**Fig. 1:**
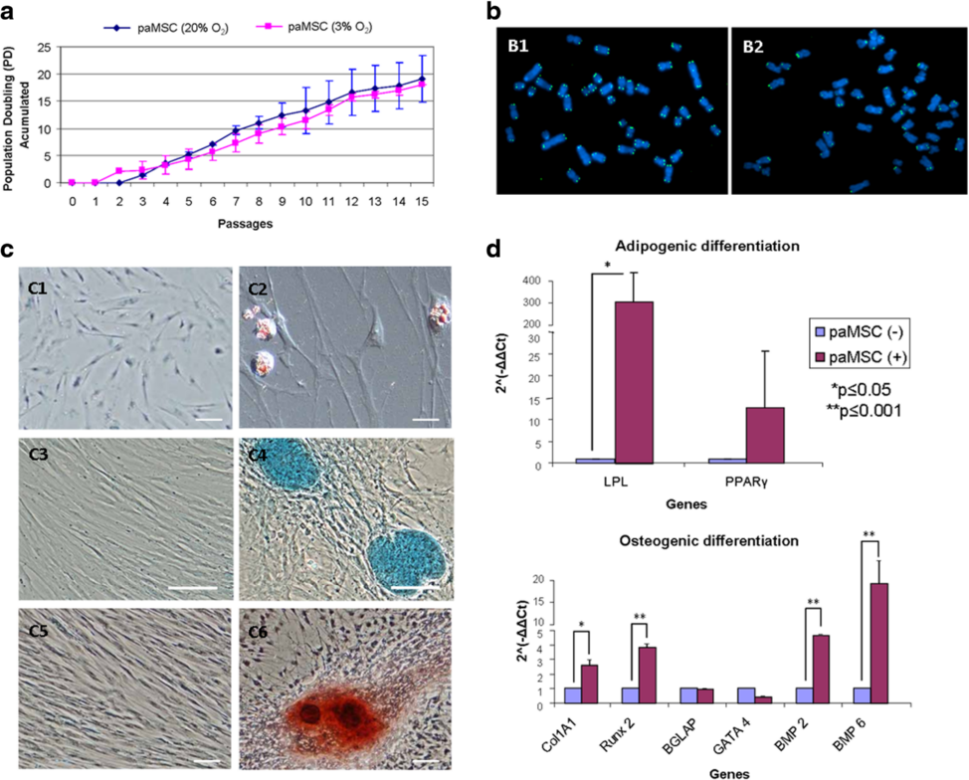
**a** Cell proliferation in distinct O_2_ conditions. Accumulated population doubling (*PD*) in a representative pig mesenchymal stem cells from adipose tissue (*paMSC*) cell isolate (*n* = 2) at low (3 % O_2_, *pink line*) and conventional oxygen tension (20 % O_2_, *blue line*). Cumulative PD was calculated with the formula PD = (log (Nn/Nn_1))/log 2 (n = passage; N = cell number). **b** Comparative karyotype in porcine and human MSC from adipose tissue. Karyotype of paMSC cultured at 20 % O_2_ (B1) compared with hMSC cultures at 20 % O_2_ (B2). Porcine karyotype (2n = 38) showing two sex chromosomes and 18 autosome pairs. **c** In vitro differentiation capacity of paMSC. Representative images showing in vitro paMSC differentiation under phase contrast and DIC (differential interference contrast) microscopy. Oil Red O (C1, C2), Alcian Blue (C3, C4), and Alizarin Red S (C5, C6) stainings of paMSC treated with control (C1, C3, C5) or adipogenic (C2), chondrogenic (C4), and osteogenic (C6) differentiation media. *Scale bars* = 100 μm and 20 μm (C2). **d** Semiquantitative RT-qPCR of marker genes in paMSC after adipogenic and osteogenic differentiation. Fold expression of adipogenic and osteogenic markers after quantitative experiments of adipogenic and osteogenic lineages (*n* = 4). ACTB was used as the endogenous gene. paMSC (–) correspond to cells grown with standard medium; paMSC (+) to cells grown in the specific differentiation medium. *p ≤ 0.05; **p ≤ 0.001

To determine whether oxygen concentration influenced paMSC growth and function, as shown for human MSC [36], we established paMSC cultures (5 × 10^3^ cells/cm^2^) and compared growth rates at 20 % and at 3 % pO_2_ over 15 passages. paMSC cultures at high and low O_2_ concentrations showed similar growth rates, which were slightly higher for cells at 20 % pO_2_ (Fig. 1a). The paMSC karyotype was similar to that of the domestic pig (2n = 38) and relatively similar to human haMSC when both were grown under 20 % pO_2_ conditions (Fig. 1b).

To demonstrate paMSC pluripotency, we performed classical differentiation studies using three mesoderm cell lineages: adipocytes, chondrocytes, and osteocytes (Fig. 1c). In addition, semiquantitative RT-PCR confirmed an increase in most analyzed genes associated with the specific differentiated cells, including peroxisome proliferator-activated receptor γ (*PPAR-γ*) and finding statistically significant differences with lipoprotein lipase (*LPL*) in adipogenic differentiation. These changes were statistically significant in the case of collagen type I alpha 1 (*COL1A1*), *RUNX-2*, and *BMP2/6* in osteogenic differentiation (Fig. 1d); *ACTB* (β-actin) was used as the reference gene.

Cellular and molecular characterization studies confirmed the similarity of porcine MSC with human and murine MSC [37–39], and our unpublished results. The studies suggested that paMSC growth is more resistant to oxidative stress than such cells in other species.

### Genetic manipulation of paMSC for IGF-1 or HGF overexpression

Our main aim was to test the effect of sustained IGF-1 and HGF co-administration in an in vivo porcine infarction model. We used pRRLsin18.CMV-IGF-1-IRES-GFP (paMSC-IGF-1-GFP) and pRRLsin18.CMV-HGF-IRES-Cherry (paMSC-HGF-Cherry) lentiviral vectors (see Additional file 3: Figure S1A) to transduce paMSC, thus inducing co-expression of GFP and IGF-1 or Cherry and HGF, respectively. paMSC transduction was optimized with the empty control vector pRRLsin18.CMV-IRES-GFP (gfp) for effective expression without inducing apparent deleterious effects. Transduced paMSC, paMSC-IGF-1-GFP (see Additional file 3: Figure S1B), in general referred to as paMSC-mod, showed a similar behavior and were easily purified by cell sorting (>90 %); an MOI of 50 was used for further work. No influence of pO_2_ on either transduction efficiency or the subsequent paMSC-GFP sorting and expansion were observed (see Additional file 3: Figure S1C).

**Fig. 2:**
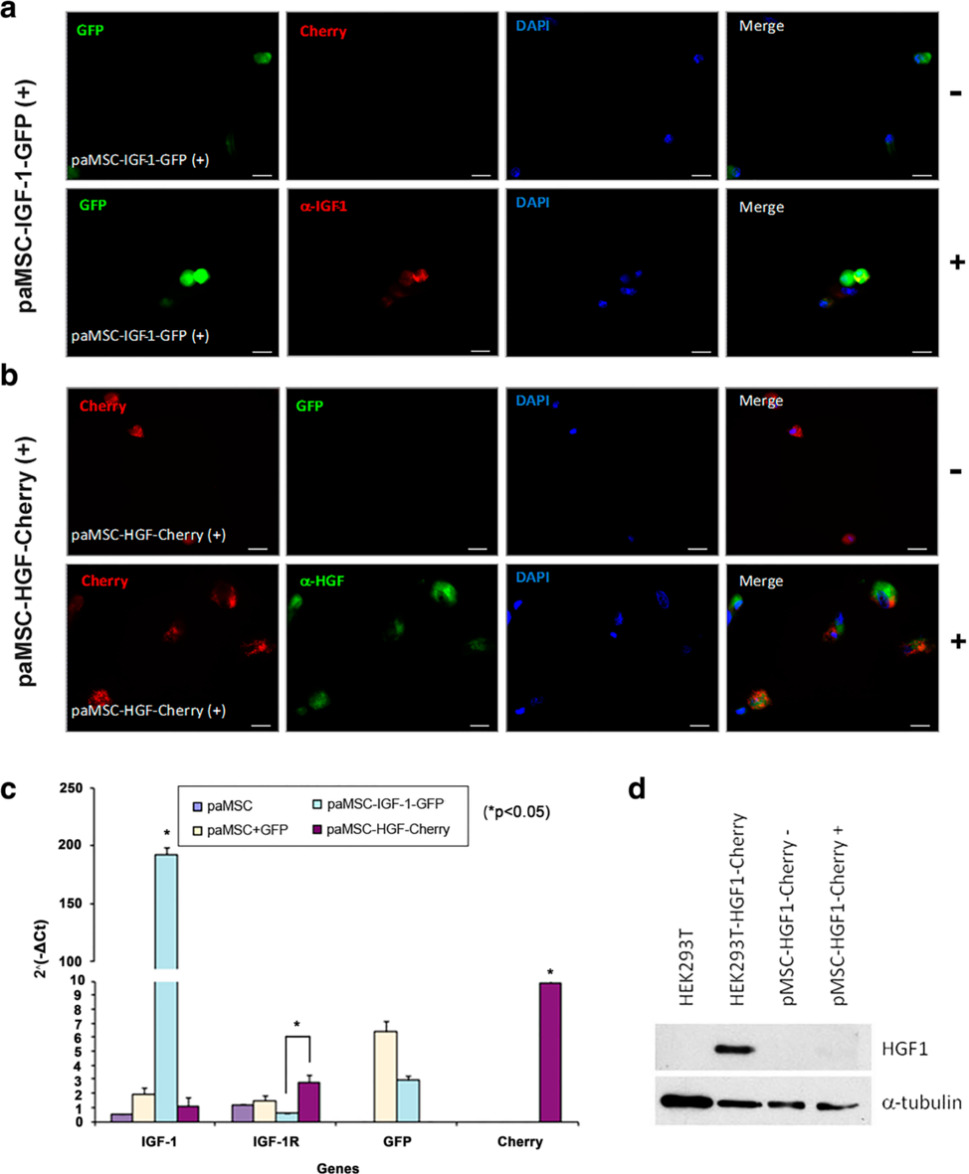
Insulin-like growth factor-1 (*IGF-1*) and hepatocyte growth factor (*HGF*) overexpression in pig mesenchymal stem cells from adipose tissue (*paMSC*) after lentiviral transduction. **a** α-IGF-1 immunocytochemistry in paMSC transduced with the pRRLsin18.CMV-IGF-1-IRES-GFP lentiviral vector (positive fractions). Negative controls (–) show evaluation of Cherry + cells; *scale bars* = 20 μm. **b** α-HGF immunocytochemistry in paMSC transduced with the pRRLsin18.CMV-HGF-IRES-Cherry lentiviral vector (positive fractions); analysis was performed after cell separation. Negative controls (–) show evaluation of green fluorescent protein (*GFP*) + cells; nuclei were DAPI-stained; *scale bars* = 20 μm. **c** Relative expression of selected markers and genes to characterize paMSC-mod cells. IGF-1, IGF-1R, GFP, and Cherry expression relative to the endogenous *GusB* gene in the cell populations (*n* = 2; *p ≤ 0.05). **d** Western blot analysis of HGF in several cell populations transduced with the pRRLsin-18.CMV-HGF-IRES-Cherry vector. HEK293T cell lines were used as a control; α-tubulin was used as a loading control

MSC manipulation was monitored by comparison with transduced HEK293 cells (control) as a reference. paMSC-IGF-1-GFP cells showed a specific increase in IGF-1 expression (see Additional file 4: Figure S2A-Vi) with basal HGF expression (see Additional file 4: Figure S2B-ii(MSC)). paMSC-HGF-Cherry cells showed specific enhancement of HGF expression (see Additional file 4: Figure S2B-Vi), with no increase in IGF-1 expression (see Additional file 4: Figure S2A-ii(MSC)). paMSC-IGF-1-GFP and paMSC-HGF-Cherry cultures were purified, and IGF-1 and HGF expression monitored by immunocytochemical staining for markers and controls in positive- and negative-sorted fractions (Fig. 2a and b; see Additional file 5: Figure S3); Fig. 2a shows the GFP-positive (+) fraction obtained after paMSC-IGF-1-GFP sorting, with analysis of the GFP-negative (–) fraction (see Additional file 5: Figure S3A). The results obtained were similar to those of paMSC-HGF-Cherry cells, with analysis of the Cherry-positive (+) fraction, which showed enhanced HGF expression (Fig. 2b) and of the Cherry-negative (–) fraction, which demonstrated basal HGF levels (see Additional file 5: Figure S3B). Comparative analysis of paMSC-IGF-1-GFP cells with unmodified paMSC, paMSC transduced with empty vector (paMSC-GFP), and paMSC-HGF-Cherry cells showed a significant IGF-1 overexpression that correlated with GFP expression (*p* ≤ 0.05). paMSC-HGF-Cherry cells showed basal IGF-1 levels and significant differences in IGF-1R levels compared with paMSC-IGF-1-GFP (*p* ≤ 0.05) (Fig. 2c). Reverse analysis showed similar results; no alterations were found in *c-MET* (HGF receptor) expression in any cell population (not shown). Western blot analysis confirmed weak but clear HGF overexpression in paMSC-HGF-Cherry cells (Fig. 2d), but did not confirm IGF-1 expression, probably due to inappropriate antibodies for the pig (not shown). Results indicated that IGF-1 is selectively overexpressed in paMSC-IGF-1-GFP; we also observed a significant reduction (*p* ≤ 0.05) in the IGF-1 receptor (Fig. 2c).

**Fig. 3:**
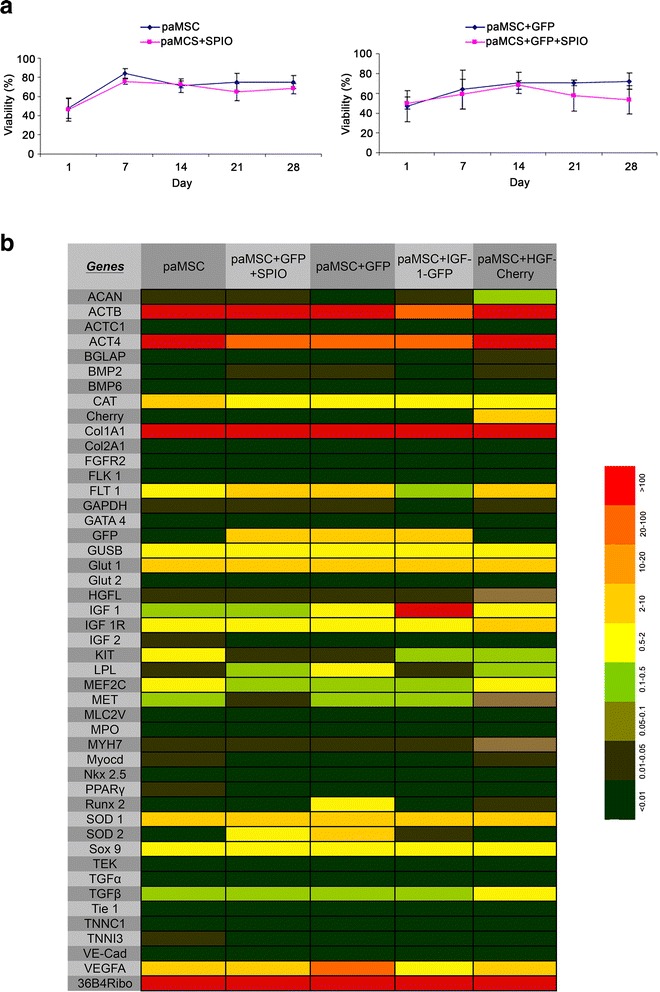
**a** Effect of superparamagnetic iron oxide (*SPIO*) labeling in pig mesenchymal stem cells from adipose tissue (*paMSC*) viability. paMSC were transduced with pRRLsin.18.CMV-IRES-GFP or were mock-transduced. Both populations were SPIO-labeled (day 0), followed by weekly trypan blue exclusion analysis for 1 month. paMSC mock-transduced (*left panel*) or paMSC-transduced cells (*right panel*); SPIO-labeled cells (*pink lines*) in comparison with mock cells (*blue lines*). Results are shown as viability (%). **b** RT-qPCR gene expression profile of transduced paMSC. paMSC populations were analyzed by RT-qPCR at similar passages (*n* = 2). For those used in the in vivo evaluation, results are shown as the mean of all values. Color scale indicates relative expression values

paMSC viability after labeling for in vivo MRI detection was tested by cell count analysis using Trypan Blue, which showed no differences between unlabeled and SPIO-labeled cells in any subpopulation (Fig. 3a). Furthermore, to confirm maintenance of basal gene expression profiles after expansion, we used RT-qPCR to study a selected panel of genes in all paMSC-mod cells and controls immediately before transplant (Fig. 3b). Although some variation was observed, we confirmed much higher *IGF-1* expression in paMSC-IGF-1-GFP cells (*p* ≤ 0.0001). As in paMSC-GFP cells, this group also showed GFP expression. paMSC-HGF-Cherry cells showed higher Cherry expression, absent in the remaining populations. *ACAN* (aggrecan), *MYH7* (myosin heavy chain 7), *MEF2C* (Myocyte Enhancer Factor 2C) (*p* ≤ 0.05) and *HGFL* (Hepatocyte Growth Factor-Like Protein) levels were increased compared with other populations. Only small differences were found in expression of the primitive cell marker *KIT*, with higher levels in non-transduced cells than in cells transduced with IGF-1 or HGF; paMSC-GFP cells expressed lower *KIT* levels. *RUNX2*, *VEGFA* (*p* ≤ 0.0001), *LPL* and *SOD2* levels were also increased in paMSC-GFP cells (Fig. 3b).

### Effects of paMSC-mod transplantation in a porcine model of AMI

**Fig. 4:**
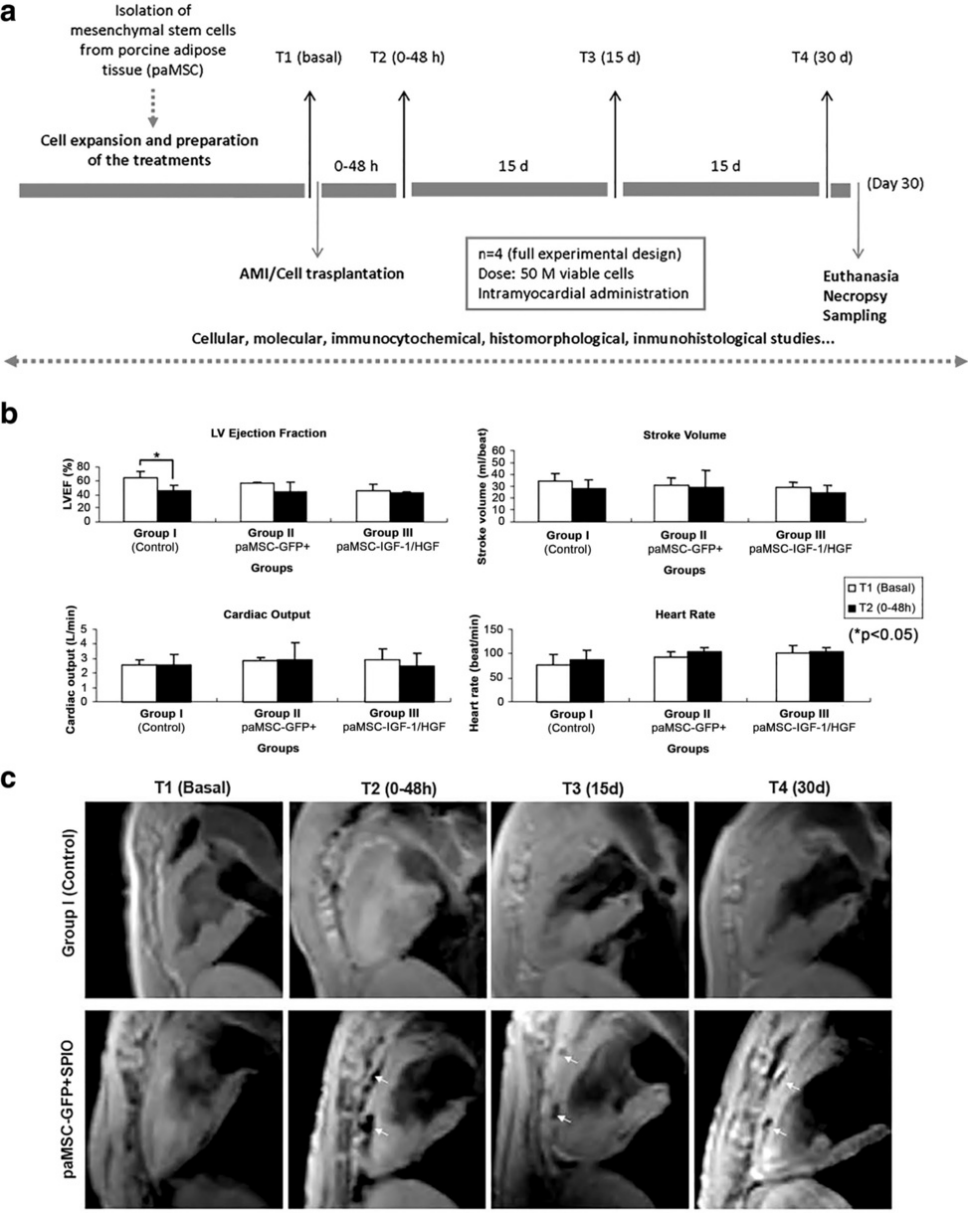
**a** Experimental design for the in vivo study. *Black arrows* indicate MRI monitoring, at which time blood samples were obtained for analytical determinations. **b** T1 vs T2 cardiac function studies. Analysis of cardiac function parameters (left ventricular ejection fraction (*LVEF*), cardiac output (CO), stroke volume (SV) and heart rate (HR)), comparing T1 and T2 for each group (*p ≤0.05). **c** Cell localization by MRI. T2-star CMR representative images in a long-axis view of a heart that received paMSC-GFP + SPIO cells vs the control group, at different time points. *T1*, baseline before cell transplant; *T2*, 0–48-h follow-up; *T3*, 15 days; *T4*, 30 days. *White arrows* indicate SPIO-labeled cells. *AMI* acute myocardial infarction, *GFP* green fluorescent protein, *HGF* hepatocyte growth factor, *IGF-1* insulin-like growth factor-1, *paMSC* pig mesenchymal stem cells from adipose tissue

Using an in vivo porcine myocardial infarction model, we analyzed the effectiveness for cardiac repair of heterologous paMSC previously transduced for HGF or IGF-1 overexpression (paMSC-mod). Figure 4a summarizes the overall experimental design used to compare the potential therapeutic effects of paMSC-mod in vivo in three experimental groups: group I (control), group II (paMSC-GFP), and group III (paMSC-IGF-1 and -HGF).

Infarct size was estimated in all groups 48 h post-AMI (see Additional file 6: Figure S4A). Plasma levels of the infarct markers TnI (troponin) and myoglobin were clearly increased from T1 to T2 in all treatment groups compared with controls, with a maximum in group III of 20 μg/l TnI (see Additional file 6: Figure S4B) and 250 μg/l myoglobin (see Additional file 6: Figure S4C). These T2 plasma levels were reduced at T4 to nearly basal levels (data not shown). Plasma levels of the infarct marker CKMB varied greatly among groups, even at T1 (see Additional file 6: Figure S4D) and at the 1-month (T4) follow-up.

Functional measurements revealed that LVEF was decreased significantly (*p* ≤ 0.05) 48 h post-AMI with respect to baseline levels. In addition, stroke volume inversely correlated with heart rate evolution within all groups and cardiac output was not altered between groups (Fig. 4b). These data suggested that all animals in each experimental group sustained similar cardiac damage and that differences were evident by 48 h after AMI induction. Furthermore, a significant retention of paMSC-mod cells was demonstrated by cardiac MRI. Figure 4c shows the long-axis view of T2-star CMR images of a control-MI representative animal and another similar to group II, paMSC-GFP-transplanted, but labeled with SPIO. paMSC engraftment within the infarcted myocardium were observed at any analyzed time-point, confirming cell retention and survival, 1 month after transplant.

**Fig. 5:**
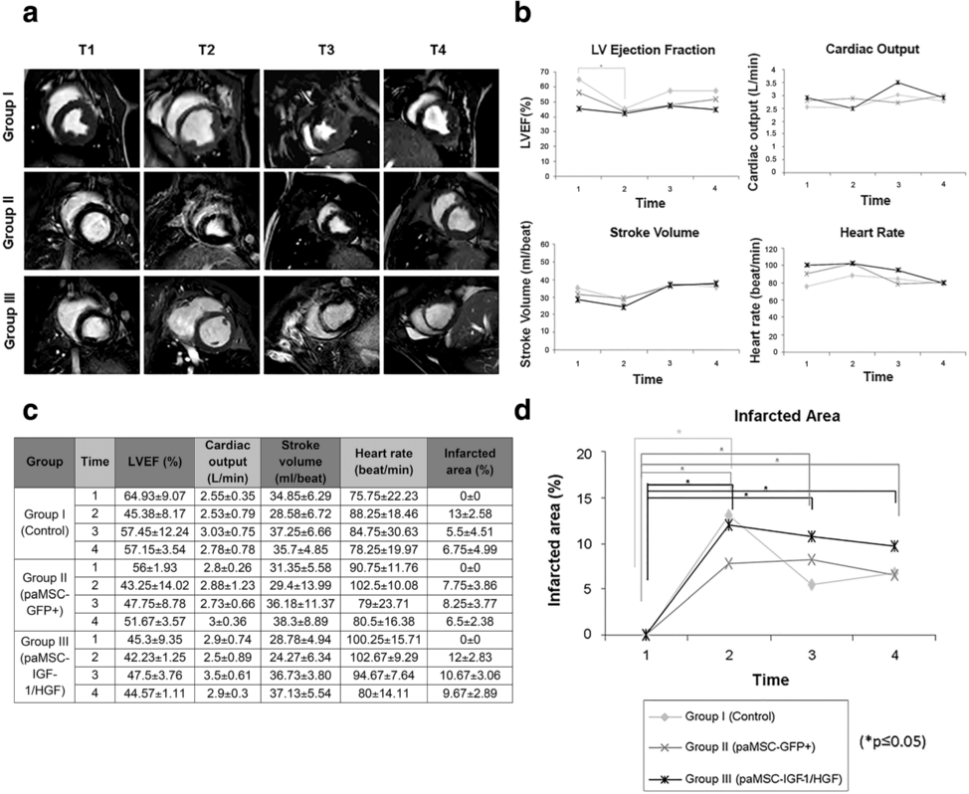
AMI evolution monitored by MRI. **a** Image sequence for a representative animal from each study group at the four follow-up times. Images were taken on the short axis in the T2 format. **b** Cardiac function studies. Left ventricle ejection fraction (*LVEF*), cardiac output (CO), stroke volume (SV), and heart rate (beats/minute) were analyzed. **c** Numerical data of cardiac function parameters and infarcted area. **d** Infarcted area evaluation. This graph shows the results obtained (%) after analyzing the infarcted area by MR images during a month of monitoring. On the *x* axis for **b** and **c**, 1, 2, 3, and 4 indicate follow-up times as described in Fig. 4c (*p ≤ 0.05). *GFP* green fluorescent protein, *HGF* hepatocyte growth factor, *IGF-1* insulin-like growth factor-1, *paMSC* pig mesenchymal stem cells from adipose tissue

T2 short-axis delayed enhancement images summarizing infarct size evolution for all groups are shown in Fig. 5a. Images from the first 15 days post-AMI induction (T2 and T3) show gradual reduction of the IVS closer to the apex, mainly in the control group and group III, with clear reduction of the ventricular wall. At 1 month (T4), although recovery from lesions was not complete, all groups showed a slight improvement in affected areas, with images similar to baseline in some cases (control group and group II).

After treatment, parameters improved over time, with similar levels in treated groups. At 1 month, the percentage of recovery, calculated by comparing the LVEF in T4 with T2, was higher in controls (group I, 11.77 %) and group II (8.43 %) than in group III (2.34 %) (Fig. 5b). Comparison of cardiac output and stroke volume showed some similarities (Fig. 5b). The control group showed a reduction in both values in T2 relative to T1, which improved relative to basal values in T3. In the last 15 days of the study, these values stabilized and decreased slightly, but were still above baseline levels (T1); Fig. 5c shows mean values for all groups at all times of analysis (T1–T4). The evolution of cardiac output and stroke volume for group II (paMSC-GFP-injected) was generally similar to the other groups throughout the study, with a difference in T4 in which values improved further compared to T3. The evolution of cardiac output was similar in group III (paMSC-IGF-1/HGF) to the other groups, with improved stroke volume in T4 compared to T3, as for group II. Heart rate values varied considerably in all treated groups throughout the experiment, although it was generally lower after 1 month (T4) compared to basal levels (T1) (Fig. 5b); this parameter increased moderately in controls (group I), with slight differences compared to basal levels. Although statistical analysis showed no significant differences in any of the parameters analyzed, MRI allowed straightforward follow-up of changes in cardiac pathology for all groups (Fig. 5c).

Infarcted area measurements after AMI showed values for damaged areas at T2 ranging from 7.8 to 13 %, with the largest infarcts in the control group and group III (Fig. 5d). All groups showed a gradual reduction in the percentage of infarcted areas. Kinetics and reduction of infarcted area were similar in group II and the control group (~5 %); recovery kinetics was slower in group III animals, which maintained a larger infarcted area (9.7 %) than other groups (Fig. 5d).

**Fig. 6:**
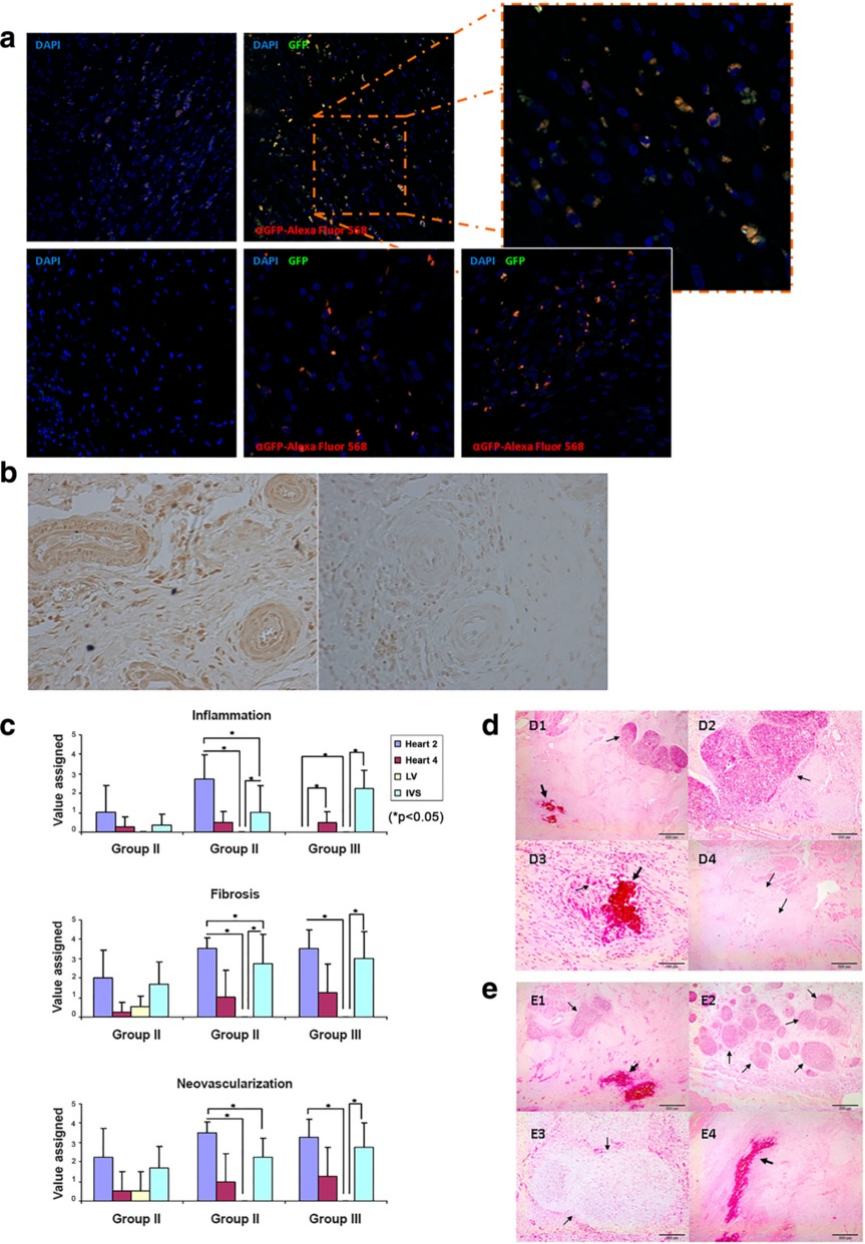
**a** Immunohistochemical detection of GFP+ paMSC in heart sections. Heart sections were analyzed from two group III pigs (Heart 2 section) using Alexa 568-labeled anti-GFP (*red*) and DAPI (*blue*) counterstaining. *Left*, negative controls (DAPI alone) to establish tissue autofluorescence. *Inset*, enlarged image from one animal (*dotted area*). **b** IGF-1 expression in myocardial sections of a representative group III pig. *Left*, anti-IGF-1 staining developed with DAB immunohistochemistry in a representative section from a group III pig (paMSC-IGF- 1-GFP/HGF-Cherry). *Right*, negative control (no anti-IGF-1). **c** Graphical representation of pathological studies on inflammation, fibrosis, and new vessel formation in the collection of samples analyzed in all experimental groups studied. Values shown as the mean of various sections for each animal in each group (*n* = 4 animals/group; *p ≤0.05) (for criteria, see Methods). **d** Histological evaluation by H/E staining in group II pigs (paMSC-GFP+). Fibrosis is very marked in this group, with calcification and granulomatous areas. (D1, D2) Heart 2 section, animal 2; (D3) Heart 2 section, animal 1, in which we observed dystrophic calcifications surrounded by multinucleated giant cells. (D4) IVS section, animal 1 showed severe fibrosis accompanied by neovascularization. (D1) *Thick arrow*: area of calcification; *thin arrow*: lymphoplasmacytic granuloma; (D2) lymphoplasmacytic granuloma; (D3) *thick arrow*: area of calcification; *thin arrow*: giant cells; (D4) *Arrows* shows fibrosis. **e** Histological evaluation by H/E staining in group III pigs (paMSC-IGF-1/HGF). (E1, E2) Heart 2 section, animal 2 showed numerous granulomas with calcification in the fibrotic area. (E3) IVS section; animal 2 showed cartilaginous metaplasia. (E4) IVS section, animal 3, large areas of calcification and a severe fibrosis. (E1) *Thick arrow*: area of calcification; *thin arrow*: granuloma. Around fibrosis; (E2) *arrows* show granuloma; (E3) *arrows* indicate the cartilaginous metaplasia; (E4) *thick arrow*: area of calcification. The rest signals were assigned to fibrotic areas. *GFP* green fluorescent protein, *HGF* hepatocyte growth factor, *IGF-1* insulin-like growth factor-1, *IVS* interventricular septum, *LV* left ventricular, *paMSC* pig mesenchymal stem cells from adipose tissue

As treatment of infarcted pigs with IGF-1- and HGF-overexpressing paMSC had no apparent effect on functional recovery, we tested for paMSC-GFP+ cells in post-euthanasia samples (T4). In a positive control animal (paMSC-GFP + SPIO transplanted but not infarcted), GFP+ cells were present and stained yellow-orange (see Additional file 7: Figure S5A), with no signal in negative controls (see Additional file 7: Figure S5B). We evaluated these cells in samples from animals from each group and observed GFP+ cells in group II and III (Fig. 6a; see Additional file 8: Figure S6); these cells appeared in clumps and were not isolated, as in the control transplanted, no infarcted pig (see Additional file 7: Figure S5A and see Additional file 9: Figure S7B). Molecular studies (genomic PCR) of cardiac tissue showed GFP-positive cells in a Heart 2 sample from the infarcted animal with paMSC-GFP, labeled with SPIO (see Additional file 9: Figure S7A). In addition, we also confirmed IGF-1 overexpression (detected by DAB immunohistochemistry) in group III samples (Fig. 6b).

### Evaluation of inflammation, fibrosis, and neovascularization in paMSC-mod-transplanted pigs after AMI

Results derived from anatomopathological evaluation of myocardial sections are summarized in Fig. 6c. Samples from myocardium, Heart 2, Heart 4, LV, and IVS of animals from each group (*n* = 4) are shown. LV samples were used as the reference control in each animal, as this remote myocardial region is not damaged by infarction or cell transplant. Statistical analyses showed no significant differences in each section among groups (Kruskal-Wallis test); comparison, however, of all sections within each group (Mann-Whitney test) indicated significant differences in all parameters analyzed. In this figure it should be noted that a moderate-low or negative (group III) inflammation was found in Heart 2 samples, while the rest of the sections analyzed showed similar levels to the other groups (i.e., IVS; group III). This is the clearest difference between groups II and III. Fibrosis events (see the score definition in the Methods section) were found in groups II and III; these were clearest in Heart 2 sections, followed by IVS sections, and negative in LV (Fig. 6c).

Large dystrophic calcification areas were observed in three animals, two in Heart 2 sections and one in IVS (group II; Fig. 6D1 and D3). Group III pigs had more damaged areas than other groups, especially fibrosis in Heart 2 and IVS samples. Fibrosis was pronounced in Heart 2 sections from three of four animals and two of four IVS sections; it was moderate in other sections, and mild in one (LV). This group showed fibrosis even in the Heart 4 section, whereas it was barely observed in Heart 4 samples from most animals in other groups (only group II, animal 1).

Interestingly, groups II and III demonstrated an increased and similar (almost parallel) pattern of fibrosis and neovascularization in the different regions analyzed, especially evident in Heart 2 samples. This correlation was less clear for inflammation, although group III animals showed multifocal granulomas (Fig. 6E2) that were larger in fibrotic areas, with many new blood vessels; group III animal 2 also had moderately sized dystrophic calcification areas (Fig. 6E1) and cartilaginous metaplasia areas in IVS (Fig. 6E3).

Overexpression of IGF-1 and HGF via paMSC promoted neoangiogenesis and reduced inflammation in some heart sectors, although they were not proportional to values for cardiac function recovery.

## Discussion

Adipose tissue-derived MSC (haMSC) are considered a promising stem cell type given the abundance of stem cells in this tissue, which has no donor limitation and is easily available by low-invasive methods [40]. Results in AMI treatment are modest, however, and the mechanisms involved are still not fully understood [40, 41]. Recent studies using two distinct cardiac stem cell (CSC) populations showed encouraging results in early clinical evaluation [16–18].

Survival, engraftment, and persistence of transplanted cells or their progeny is extremely limited [42]. The frequently reported moderate improvement in cardiac function is thought to be produced by the liberation of paracrine factors that mediate survival, neovascularization, remodeling, and cell proliferation [43, 44]. Exosomes were also shown to mediate many MSC functions [45], later observed in CSC [46]. Analysis of the angiogenic potential of MSC-secreted factors (conditioned medium) for direct therapeutic use indicated a reduction in infarct size and conservation of systolic and diastolic cardiac output, which confirmed the value of these factors in counteracting AMI. Finally, other studies have also demonstrated the immunomodulatory properties of MSC that are related to their capacity to migrate to injury sites and/or neovascularize in ischemic areas, acting on different subsets of immune cells [47]; this property has been potentiated using genetically modified MSC [48].

Our initial studies of paMSC growth established appropriate conditions to obtain adequate cell doses for treatments. Proliferation studies showed distinct behavior of paMSC compared with human MSC grown at different oxygen concentrations. In low oxygen conditions, human MSC cultures showed significantly greater genetic stability and higher yields [36, 49]; this was related to elevated oxidative stress and DNA damage caused by growth at high oxygen tension, which helped to accelerate senescence [36, 50]. Results in the porcine model showed a similar paMSC growth profile at low and high oxygen tension, which suggests greater paMSC resistance to oxidative stress than with human MSC. Genetic stability of paMSC was confirmed in both oxygen conditions [51], and cells maintained multipotent differentiation capacity. Some differences were nonetheless observed in comparison with haMSC, which suggested intrinsic biological differences that could affect the therapeutic responses of paMSC vs human MSC.

Post-AMI stimuli activate CSC mediated by paracrine feedback between myocytes and the CSC. In response to stress, myocytes produce growth factors and cytokines for which CSC have receptors [25]. After demonstrating that CSC respond to growth factors secreted by adjacent myocytes, Urbanek et al. confirmed the efficiency of combined recombinant IGF-1/HGF treatment in mice and dogs [52]. CSC are activated in situ by local administration of IGF-1/HGF in a porcine heart infarction model [53], which improved ventricular function in pigs. Therefore, intracoronary administration of these factors was proposed as a strategy to reduce post-AMI cardiac remodeling and induce cardiac regeneration [25]. Finally, direct IGF-1 + HGF administration was recently evaluated in a porcine model of chronic myocardial infarction (MI), in which growth factor delivery reduced pathological hypertrophy, led to formation of new small cardiomyocytes, and increased capillarization [54].

Priming (preconditioning) of MSC or co-administration with growth factors is also used to augment therapeutic potential. An IGF-1 + HGF combination loaded in polylactic-co-glycolic acid microcarriers with haMSC enhanced engraftment of the transplanted haMSC cells and showed a 1.3-fold higher density of medium-sized blood vessels in the infarct border zone [29]. hMSC preconditioning with IGF-1 prior to transplant in infarcted rats increased engrafted cell survival in the ischemic heart, decreased myocardium cell apoptosis, and reduced inflammatory cytokines [55]. In most preconditioning strategies, IGF-1 is also proposed as a mediator [56].

IGF-1 and HGF are thus being evaluated in different modalities of cardiac repair [43, 57], although the mechanisms involved remain to be fully understood. Some studies showed that HGF treatment post-AMI attenuates systolic cardiac remodeling and cardiac dysfunction, with a cardioprotective effect; these effects were linked to angiogenic and anti-apoptotic mechanisms [58]. Evidence also implicates IGF-1 in vascular protection, which might be beneficial in chronic cardiac insufficiency [59] and in treatment of sepsis-associated cardiac dysfunction [60].

Given the promising results with direct HGF and/or IGF-1 administration [25, 54], several attempts have been made to engineer MSC to vehiculate IGF-1 or HGF expression. Kouroupi et al. manipulated neural stem/precursor cells (NPC) to overexpress IGF-1; using live-imaging techniques, they reported that IGF-1 transduction enhanced the motility and tissue penetration of grafted NPC [61], although no significant in vivo improvement was demonstrated [62]. Human MSC and paMSC were transduced with lentiviral vectors to overexpress IGF-1 [63]; overexpression of this gene improved induction by 5-azacytidine and promoted limited cardiomyocyte-like differentiation [63]. Experience is broader for genetic manipulation of MSC to overexpress HGF. Early work with rat BM-MSC showed decreased infarcted scar area and increased angiogenesis in HGF-MSC-treated animals [64]. In the porcine model, paMSC (alone or vascular endothelial growth factor (VEGF)/HGF-transfected) improved cardiac function and perfusion, probably by increasing angiogenesis and reducing fibrosis; MSC + HGF was superior to MSC + VEGF [65].

We used the porcine model to explore the synergistic effect of combined, sustained administration of paMSC modified individually to overexpress IGF-1 and HFG, labeled with fluorescent markers. This approach would allow later adjustment of the balance between growth factor supply by altering the ratio of the two paMSC populations. We generated and validated optimized lentiviral vectors and transduced paMSC, followed by purification, by which we obtained enriched paMSC-IGF-1-GFP and paMSC-HGF-Cherry cells that were evaluated in vitro and in vivo. Results showed that the cell doses used in the animals caused no toxicity or short-term safety problems.

We found improvement in cardiac function (increases in LVEF, cardiac output, and stroke volume, and reduction in heart rate and infarction area) in all groups from day 15 (T3), although these changes were not statistically significant in any case and were similar to non-paMSC-treated infarcted controls (group I). Although intragroup variability was marked, it did not appear to be the main reason for these results. Overall results for treatment group III pigs (paMSC IGF/HGF) were in fact poorer than those for group II (paMSC-GFP; paMSC bearing the empty vector). At termination of the in vivo experiment, the heart apex and IVS showed fibrotic areas during macroscopic assessment and sampling, and evaluation of infarction degree by Masson’s trichrome staining showed a larger proportion of affected areas in group III (see Additional file 10: Figure S8), and these pigs had larger infarctions than in other groups.

Although paMSC could not be identified in all samples, immunohistochemistry and molecular assays confirmed live paMSC in some group II and III tissues at 1 month (T4) follow-up, which suggests that the functional results are not due to elimination of paMSC-IGF- 1/HGF. Comparable results have been reported after autologous porcine BM-MSC implant for treatment of aortic injury [66].

Hematoxylin/eosin staining was used to evaluate the degree of resolution of cardiac damage after each treatment. The control group (I) had the lowest degree of damage, mainly in Heart 2 and IVS samples, but pericarditis was more severe compared with other groups and lesions were visible in all sections. By contrast, group III Heart 2 and IVS sections were the most affected by fibrosis and inflammation, followed by those of group II. Angiogenesis was also more evident in sections from group III than the other groups. We also found a correlation between fibrosis and neovascularization, especially in groups II and III.

Several factors could contribute to the lack of significant differences. Kren et al. showed accelerated healing and repair kinetics in young pig models of AMI reperfusion like ours [67], which would limit the length of the experimental window. In addition, high intragroup variability and a reduced number of animals per group are study limitations, especially in the case of negative results.

The time of paMSC transplant also influences effectiveness; the optimal range varies from the time of AMI to 1 week later, although a specific suitable time point remains to be established [68]. Beneficial effects have been reported following in vivo transfer of modified MSC or recombinant factors from 1 week to 1 month post-infarct induction. In addition, recent studies have shown that a large proportion of the injected cells are lost from the myocardium within the first few minutes post-injection and not more than 0.1–15 % are retained.

Although positive therapeutic results are reported for IGF-1- and HGF-expressing MSC individually, we conclude that co-administration of paMSC that overexpress IGF-1 and HGF does not appear to have a synergistic effect or promote effective cardiac repair. This could be caused by interference from high local levels of either factor. The positive correlation of enhanced neovascularization and fibrosis in a number of paMSC-IGF-1/HGF-treated pigs suggests that sustained exposure to high HGF + IGF-1 levels promotes both beneficial and deleterious effects, with no regenerative advantage. In any case, global results strongly suggest that delivery of growth factor by implantation of biodegradable microparticles in the affected area is superior to the transplant of paMSC-mod cells that probably are not efficiently retained. The consequence should be a much less efficient local supply of growth factor, with reduced increment in a therapeutic index. In addition, in case of a significant therapeutic effect using modified MSC, it will be mandatory to eliminate the possibility that non-retained MSC could find other propitious niches and favor other pathological conditions [69, 70].

## Conclusions

Taken together, this study reports that co-administration of IGF-1- and HGF-overexpressing paMSC do not promote an effective repair, although we found an enhancement of neovascularization and fibrosis in the paMSC-IGF-1/HGF-treated group, suggesting that high IGF-1 + HGF levels promote beneficial and deleterious effects that moderately improves AMI recovery in a porcine model, although not significantly.

## Abbreviations

AMI, acute myocardial infarction; BM-MSC, mesenchymal stem cells from bone marrow; CKMB, myocardial creatinine kinase; CSC/CPC, cardiac stem/progenitor cells; FACS, fluorescence-activated cell system; GFP, green fluorescent protein; H/E, hematoxylin/eosin; HGF, hepatocyte growth factor; HRP, horseradish peroxidase; IGF-1, insulin-like growth factor-1; ISCT, International Society for Cellular Therapy; IVS, interventricular septum; LVEF, left ventricular ejection fraction; MOI, multiplicity of infection; MRI, magnetic resonance imaging; MSC, mesenchymal stem cells; NPC, neural stem/precursor cells; paMSC, pig mesenchymal stem cells from adipose tissue; PBS, phosphate-buffered saline; PD population doubling; RT-PCR, reverse transcription polymerase chain reaction; SPIO, superparamagnetic iron oxide; TU, transduction units; VEGF, vascular endothelial growth factor

## Additional files

Additional file 1: Table S1. A list of the primer sequences used in this work. (DOC 94 kb) Additional file 2: Table S2. A list of the antibodies used in this work. (DOC 104 kb) Additional file 3: Figure S1. (A) Lentiviral vectors used for IGF-1 and HGF forced expression in paMSC. (B) Fluorescence of cultures at distinct viral concentrations after transduction with the pRRL-sin-IGF-1-IRES-GFP vector. Images for paMSC-IGF-1-GFP, 5 days post-transduction, at distinct multiplicity of infection (MOI, 1–50). (C) Cell sorting after paMSC transduction. Four subpopulations resulting from sorting. *Top panels*, results for a subpopulation cultured at 3 % O_2_; *bottom*, transduced paMSC cultured in 20 % O_2_ conditions. An un-transduced cell population was used as a negative control. Purity of sorted GFP-positive paMSC was 95 % and 91.1 % for cells cultured at 3 % and 20 % O_2_, respectively. (TIF 447 kb) Additional file 4: Figure S2. Immunocytochemistry analysis of IGF-1 and HGF expression in paMSC-IGF- 1-GFP (A) and paMSC-HGF-Cherry (B). (+) indicates cells transfected with pRRL-sin-IGF-1-IRES-GFP (A) or pRRL-sin-HGF-IRES-Cherry vectors (B); (–) negative controls. GFP- (A-V) or Cherry-positive cells (B-V) were tested for IGF-1 (A-Vi) or HGF (B-Vi) expression; nuclei were DAPI stained (iii and Vii), showing that all paMSC-IGF-1 and HGF were positive for GFP/Cherry and IGF-1/HGF, respectively, and that paMSC-IGF-1 and HGF are negative for HGF and IGF-1, respectively. HEK293T-derived populations were used as controls (A and B; upper panels); controls confirm that HEK-293 do not express GFP/Cherry or IGF-1/HGF, and that HEK293-IGF-1 and HEK293-HGF express GFP/Cherry and IGF-1/HGF, respectively. Alexa 488- (*green*) and Alexa 568- (*red*) conjugated secondary antibodies were used. *Scale bars* = 20 μm. (TIF 414 kb) Additional file 5: Figure S3. Immunocytochemistry analysis of IGF-1 and HGF expression post-sorting in negative populations. Post-sorting immunocytochemistry for IGF-1 in GFP-negative population (A) and Cherry-negative population (B). Images confirm that IGF-1-negative cells do not express GFP/Cherry or IGF-1, and that HGF-negative cells do not express GFP/Cherry but demonstrated basal HGF levels. (C) HGF expression was monitored in paMSC-IGF-1-GFP populations (positive and negative). Positive populations were negative for HGF expression but the negative fraction showed basal HGF levels. (D) IGF-1 expression was monitored in paMSC-HGF-Cherry (positive and negative); both were positive for IGF-1 expression showing basal levels for this growth factor. (+) corresponds to primary and secondary antibodies ICC; (–) corresponds to ICC only with secondary antibodies. Secondary antibodies were Alexa-conjugated as in Additional file 4 (Figure S2). *Scale bars* = 20 μm. (TIF 242 kb) Additional file 6: Figure S4. Evaluation of infarct area and cardiac enzymes, comparing T1 vs T2. (A) Infarct area estimation, T1 vs T2. Analysis of (B) troponin I (TnI), (C) myoglobin (MYO) and (D) myocardial creatine kinase (CKMB) of all groups confirmed porcine AMI; (**p* ≤0.05). (TIF 244 kb) Additional file 7: Figure S5. (A) Immunohistocytochemistry analysis in control animals (transplanted but not infarcted). Animal transplanted with 30 × 10^6^ paMSC + GFP + SPIO cells. *Left panels* show negative controls, incubation with Alexa 568-secondary antibody (*top*) and DAPI staining (*bottom*). *Center* and *right* images show isolated GFP-positive cells (anti-GFP-A568) in the Heart 2 section. (B) Immunohistocytochemistry analysis of control animals (no treatment). *Left* and *center* images show negative controls (DAPI staining and incubation with Alexa 568-secondary antibody). *Right* image shows negative results for GFP immunofluorescence. (TIF 1493 kb) Additional file 8: Figure S6. Anti-GFP immunohistocytochemistry. (A) GFP-positive paMSC in a representative animal of group III (paMSC-IGF- 1-GFP/paMSC-HGF-Cherry). *Bottom* panel show serial cardiac sections (5 μm) counterstained with DAPI (*left*), imaged for GFP fluorescence (*center*) or anti-GFP-stained, and revealed with Cy3-streptavidin (*right*). *Top central* image corresponds to the merge of the three images shown in the bottom panel. *Top left*, negative control (group I) to determine tissue autofluorescence, stained with DAPI and anti-GFP- biotin and revealed with Cy3-streptavidin. *Top right*, an expanded view of the indicated area in the left image. (B) Sections from Heart 4 samples from animals representative of each group were stained with anti-GFP-biotin and revealed with Cy3-streptavidin, and counterstained with DAPI. *Left top*, control (group I); *right top*, transplanted uninfarcted animal; *left bottom*, group II (paMSC-GFP+); and *right bottom*, group III (paMSC-IGF-1-GFP/paMSC-HGF-Cherry) animals. (TIF 1592 kb) Additional file 9: Figure S7. (A) Molecular analysis of cardiac tissue. At 1 month post-transplant, GFP-positive cells were monitored in cardiac tissue (Heart 2) by genomic detection of GFP sequences (see Methods); ~100 bp diagnostic fragment. *ACTB* (135-bp fragment) was used for normalization. (B) Immunohistocytochemistry of anti-GFP with amplified FITC. Representative sample (Heart 4) sections from an animal transplanted but not infarcted were stained with anti-GFP-biotin and revealed with FITC-streptavidin, and counterstained with DAPI. (TIF 664 kb) Additional file 10: Figure S8. Histomorphological study of cardiac tissue by Masson trichrome staining. Representative samples from each group, showing fibrotic areas (*light blue*) resulting from AMI (*IVS* inter-ventricular septum; *LV* left ventricle). (TIF 2164 kb)
