# Supplementary material for: Combined administration of mesenchymal stem cells overexpressing IGF-1 and HGF enhances neovascularization but moderately improves cardiac regeneration in a porcine model
Source: Stem Cell Res Ther. 2016 Jul 16;7:94. doi: 10.1186/s13287-016-0350-z (PMC4947339; doi:10.1186/s13287-016-0350-z)
Supplement: Additional file 1: Table S1. — A list of the primer sequences used in this work. (DOC 94 kb) [file 13287_2016_350_MOESM1_ESM.doc]

| **Gene** | **Sense (5’- 3’)** | **Anti-sense (5’- 3’)** |
| --- | --- | --- |
| ACAN | ATCAGTGGGGAAACATCTGG | CACTAAGCTCGGTCACACCA |
| ACTB | TGCGCAGAAAATGAGATGAG | CACCTTCACCGTTCCAGTTT |
| ACTC1 | CTTCCAACCCACCCTTCTTT | GTTGCAAGTCCTGGTCTGGT |
| ACT4 | GGGAATGGGACAAAAAGACA | CATCCCAGTTGGTGATGATG |
| BGLAP | GCCCCTCACACTGCTTGCCC | TCGGTGCCCTCCTGCTTGGA |
| BMP2 | TGCGCAGCTTCCACCACGAA | CCTGTGTCTGCTCCCGAAAGACCT |
| BMP6 | GGCGGTGACGGCTGCAGAAT | CACACGACGCGGGTGTCCAA |
| CAT | CTTGGAACATTGTACCCGCT | AGATGACCCGCAATGTTCTC |
| COL1A1 | AAGACATCCCACCAGTCACC | CAGTTCTTGATTTCGTCGCA |
| COL2A1 | TGAAAAAGGTGCTCCTGGAC | CCTTCTCATCGAATCCTCCA |
| FGFR2 | AAACACGTGGAAAAGAACGG | TCACATTGAACAGAGCCAGC |
| FLK1 | CAAAACTGTCGTGATTCCATGTC | TTCTGTTACCATCAGGAACAAACCT |
| FLT1 | GTGAAGCATCGAAAACAGCA | TAGCGGGCAGATTTCTCAGT |
| GAPDH | TGGAAGGACTCATGACCACA | AGCACCAGTAGAAGCAGGGA |
| GATA4 | AATGCCTGTGGCCTCTACAT | ACCTGCTGACGTCTTCGATT |
| GUSB | CCCCAGCGATGGACCCAGGA | TCGGCCTCGAAGGGGAGGTG |
| GLUT1 | CTTCACTGTCGTGTCGCTGT | GCTCAGGTAGGACATCCAGG |
| GLUT2 | AGACACGTTTTGGGTGTTCC | GGCTAGCAGATGCCGTAGTC |
| HGFL | GGGGACGATACTGTCCTGAA | GTCCCTCAGTGCACATCTCA |
| IGF1 | GACGCTCTTCAGTTCGTGTG | CTCCAGCCTCCTCAGATCAC |
| IGF2 | TCAGGCTAGTCTCTCCTCGG | TTGAGGGGTTCAATTTTTGG |
| IGF1R | CAGTCCTAGCACCTCCAAGC | GTCTTCGGCCACCATACAGT |
| KIT | GGCATCAGGGTGACTTCAAT | GGTGGTTGTGACATTTGCAG |
| LPL | ACCTCCTGGGATACAGCCTT | GGCTTGGAGCTTCTGCATAC |
| MEF2C | TGATCAGCAGGCAAAGATTG | AGTGAGCTGACAGGGTTGCT |
| MET | CCCAATTTCTGACTGAGGGA | TAGGACCACCAGTGGAGACC |
| MLC2V | GAAACTTAAGGGGGCAGACC | CCTCCTTGGAAAACCTCTCC |
| MPO | CCAGTCCTAGCTCTGCTGCT | ATTTGGTTCTGGCGATTCAG |
| MYH7 | ATTGCCGCCATTGGGGACCG | GCCAAAGGCCTCCAGGGCAG |
| MYOCD | TCCGAGAAGGACAAGATGCT | GGACAGGTGCCTCTCTTCTG |
| NKX2.5 | CTTCTACCCGCGTGCCTAT | CCTCTGGCTTCTCCAGCTC |
| PPARγ | ATTTACACCATGCTGGCCTC | GGGCTCCATAAAGTCACCAA |
| RUNX2 | TTACTTACACCCCGCCAGTC | TATGGAGTGCTGCTGGTCTG |
| SOD1 | TCCATGTCCATCAGTTTGGA | AGTCACATTGCCCAGGTCTC |
| SOD2 | CTTCGTCTTCCTCCTCGTTG | AAACCTATGTGGGTTGCTCG |
| SOX9 | GAAGGAGAGCGAAGAGGACA | GCTTATTCTTGCTCGAACCG |
| TEK | GATTGTCCCGAGGTCAAGAA | CCATAGGACCATACGTCGCT |
| TGFα | GGTCCCCTCGGCTGGACAGT | GCAGCTATGGGCGGGTCTGC |
| TGFß | TTAACGGGTTCAATTCTGGC | TAGTTGGTATCCAGGGCTCG |
| TIE1 | AAGTTCTGTGCAAATTGGGG | GCAGAAAATCGAGCAGGTTC |
| TNNC1 | TGATTGACGAGGTGGATGAA | GTCAAACATGCGGAAGAGGT |
| TNNI3 | ATGCCCGCGTGGACAAGGTG | CGCAGGGTGGGCCGCTTAAA |
| VE-CAD | CGTGGTGGAAACACAAGATG | TGTGTACCTGGTCTGGGTGA |
| VEGFA | ATCTTCAAGCCGTCCTGTGT | TCTCTCCTATGTGCTGGCCT |
| 36B4RIBO | TCATCCAGCAGGTGTTTGAC | CAGACATACGCTGGCAACAT |
| Cherry | CCCCGTAATGCAGAAGAAGA | TTGACCTCAGCGTCGTAGTG |
| GFP | CCACATGAAGCAGCACGAC | GTGCTCAGGTAGTGGTTG |
