# Supplementary material for: Combined administration of mesenchymal stem cells overexpressing IGF-1 and HGF enhances neovascularization but moderately improves cardiac regeneration in a porcine model
Source: Stem Cell Res Ther. 2016 Jul 16;7:94. doi: 10.1186/s13287-016-0350-z (PMC4947339; doi:10.1186/s13287-016-0350-z)
Supplement: Additional file 2: Table S2. — A list of the antibodies used in this work. (DOC 104 kb) [file 13287_2016_350_MOESM2_ESM.doc]

| **Antibody** | **Catalog nº, company** |
| --- | --- |
| anti-CD105 (FITC)-mouse monoclonal | ab53318 (Abcam, Cambridge, UK) |
| anti-CD44 (FITC)-rat monoclonal | ab19622 (Abcam, Cambridge, UK) |
| anti-CD29 (FITC)-mouse monoclonal | ab21845 (Abcam, Cambridge, UK) |
| anti-CD45 (FITC)-mouse anti-pig | MCA1222F (ABD Serotec, Oxford, UK) |
| anti-CD31 (FITC)-mouse anti-pig | MCA1746F (ABD Serotec, Oxford, UK) |
| anti-SLA Class I (FITC)-mouse anti-pig | MCA2261F (ABD Serotec, Oxford, UK) |
| anti-SLA Class II DR (FITC)-mouse anti-pig | MCA2314F (ABD Serotec, Oxford, UK) |
| anti-CD90 (FITC)-mouse anti-human | 555595 (BD Pharmingen, NJ, USA) |
| anti A2B5-mouse monoclonal | MAB312 (Chemicon, CA, USA) |
| anti-tubulin (HRP), Anti-Mouse | P0447 (DAKO, Glostrup, DK) |
| anti-GFP-rabbit polyclonal | ab290 (Abcam, Cambridge, UK) |
| anti-IGF1 (H-70)-rabbit polyclonal | sc-9013 (Santa Cruz Biotech, Santa Cruz, CA, USA) |
| anti-HGF (H-145)-rabbit polyclonal | sc-7949 (Santa Cruz Biotech, Santa Cruz, CA, USA) |
| Mouse IgG1 (FITC)-isotype control | MG101 (Caltag Laboratories, Buckingham, UK) |
| Mouse IgG2a (FITC)-isotype control | ab1281 (Abcam, Cambridge, UK) |
| Mouse IgM-λ isotype control | 550963 (BD Pharmingen, NJ, USA) |
| Rat IgG2b (FITC)-isotype control | ab37364 (Abcam, Cambridge, UK) |
| Rat IgM (FITC) Rat anti-mouse | 553408 (BD Pharmingen, NJ, USA) |
| anti-Rabbit (Alexa 488); Goat-Anti-Rabbit | A11034 (Invitrogen, IL, USA) |
| anti-Rabbit-(HRP) Goat anti-Rabbit | P0448 (DAKO, Glostrup, DK) |
| anti-Rabbit (Biotin); Goat anti-Rabbit | ab6720 (Abcam, Cambridge, UK) |
| anti-Rabbit (Alexa 568); Donkey. | A10042 (Invitrogen, IL, USA) |
| Streptavidin (Cy3) | 43-4315 (Molecular Probes, CA, USA) |
| Streptavidin (FITC) | A10042 (Invitrogen, IL, USA) |
