# Supplementary figures and images for: Combined administration of mesenchymal stem cells overexpressing IGF-1 and HGF enhances neovascularization but moderately improves cardiac regeneration in a porcine model
Source: Stem Cell Res Ther. 2016 Jul 16;7:94. doi: 10.1186/s13287-016-0350-z (PMC4947339; doi:10.1186/s13287-016-0350-z)

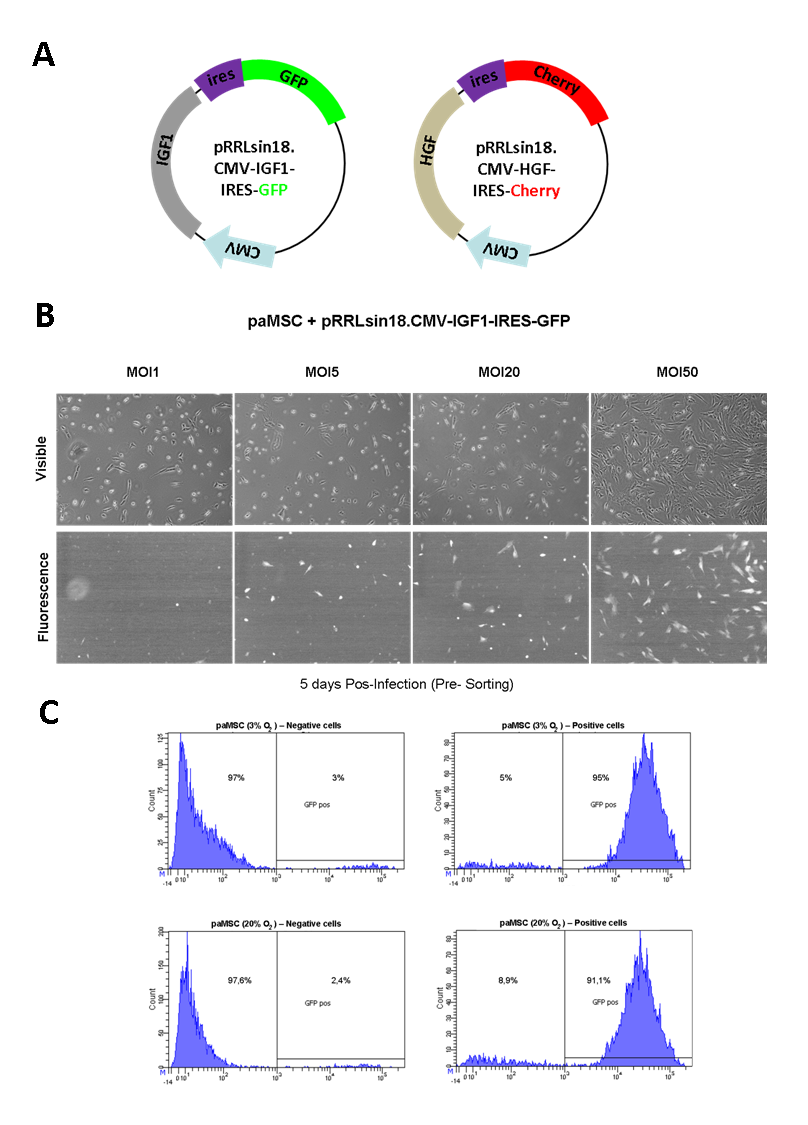

Supplement: Additional file 3: Figure S1. — (A) Lentiviral vectors used for IGF-1 and HGF forced expression in paMSC. (B) Fluorescence of cultures at distinct viral concentrations after transduction with the pRRL-sin-IGF-1-IRES-GFP vector. Images for paMSC-IGF-1-GFP, 5 days post-transduction, at distinct multiplicity of infection (MOI, 1–50). (C) Cell sorting after paMSC transduction. Four subpopulations resulting from sorting. Top panels, results for a subpopulation cultured at 3 % O2; bottom, transduced paMSC cultured in 20 % O2 conditions. An un-transduced cell population was used as a negative control. Purity of sorted GFP-positive paMSC was 95 % and 91.1 % for cells cultured at 3 % and 20 % O2, respectively. (TIF 447 kb) [file 13287_2016_350_MOESM3_ESM.tif]

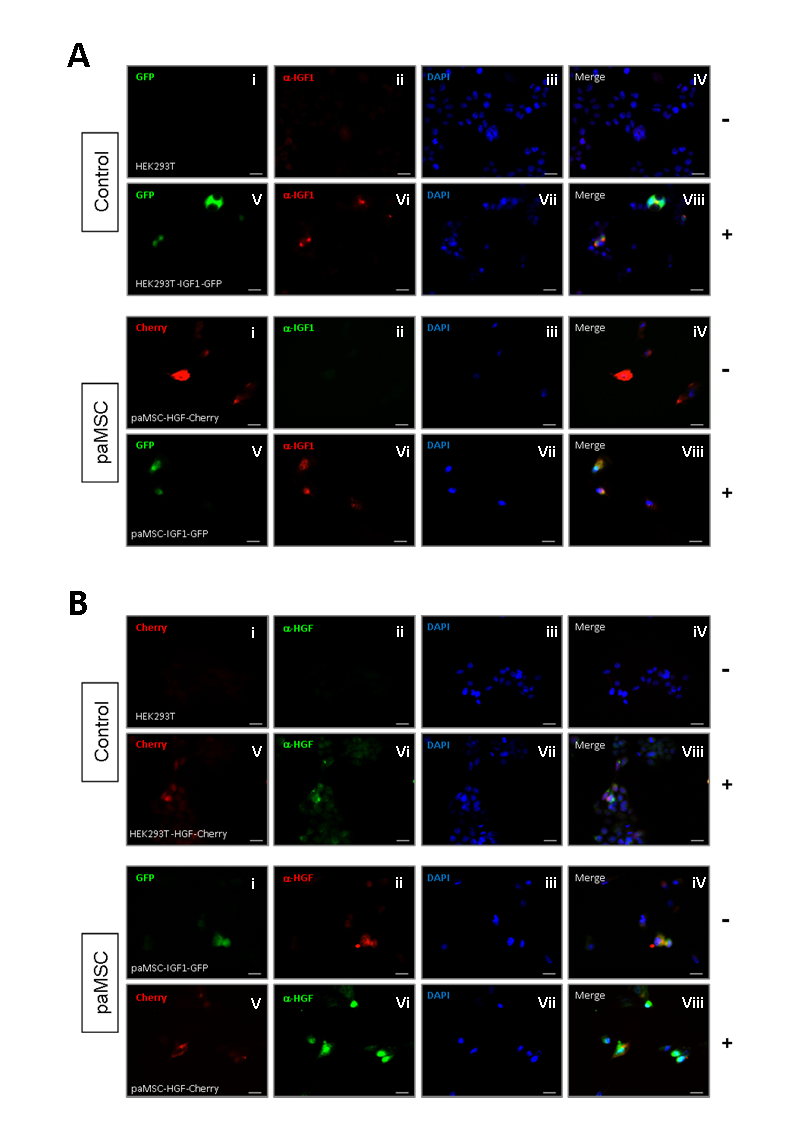

Supplement: Additional file 4: Figure S2. — Immunocytochemistry analysis of IGF-1 and HGF expression in paMSC-IGF- 1-GFP (A) and paMSC-HGF-Cherry (B). (+) indicates cells transfected with pRRL-sin-IGF-1-IRES-GFP (A) or pRRL-sin-HGF-IRES-Cherry vectors (B); (–) negative controls. GFP- (A-V) or Cherry-positive cells (B-V) were tested for IGF-1 (A-Vi) or HGF (B-Vi) expression; nuclei were DAPI stained (iii and Vii), showing that all paMSC-IGF-1 and HGF were positive for GFP/Cherry and IGF-1/HGF, respectively, and that paMSC-IGF-1 and HGF are negative for HGF and IGF-1, respectively. HEK293T-derived populations were used as controls (A and B; upper panels); controls confirm that HEK-293 do not express GFP/Cherry or IGF-1/HGF, and that HEK293-IGF-1 and HEK293-HGF express GFP/Cherry and IGF-1/HGF, respectively. Alexa 488- (green) and Alexa 568- (red) conjugated secondary antibodies were used. Scale bars = 20 μm. (TIF 414 kb) [file 13287_2016_350_MOESM4_ESM.tif]

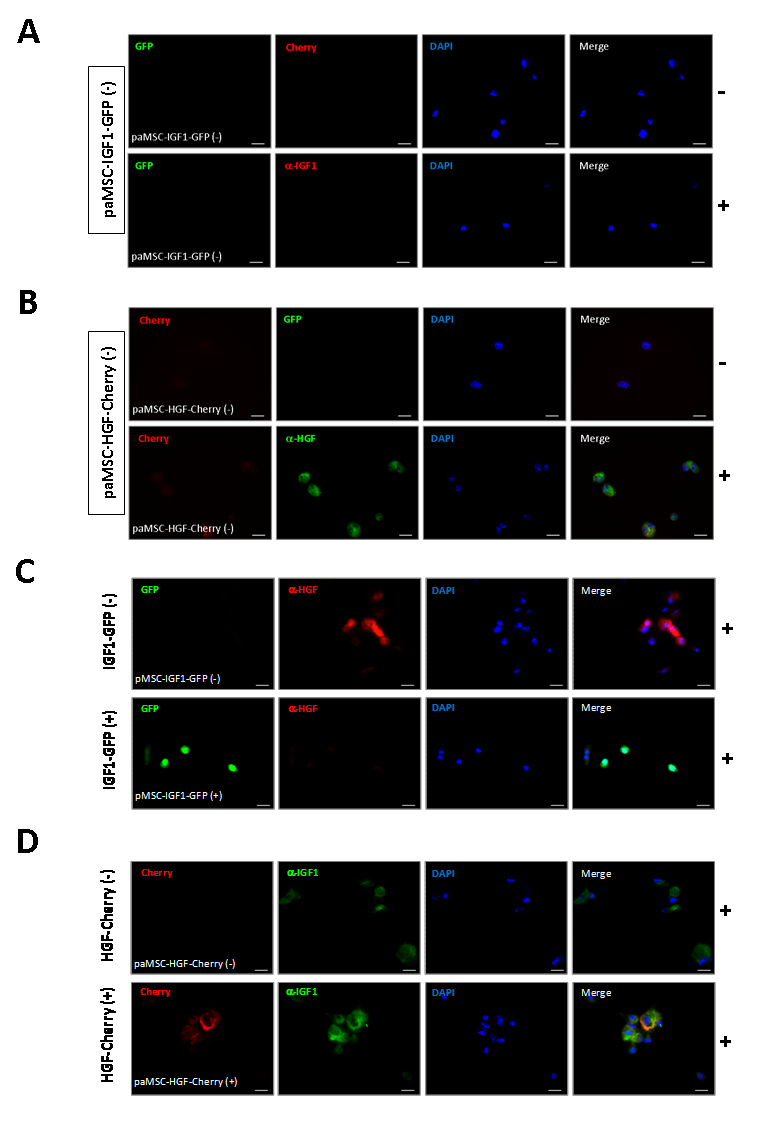

Supplement: Additional file 5: Figure S3. — Immunocytochemistry analysis of IGF-1 and HGF expression post-sorting in negative populations. Post-sorting immunocytochemistry for IGF-1 in GFP-negative population (A) and Cherry-negative population (B). Images confirm that IGF-1-negative cells do not express GFP/Cherry or IGF-1, and that HGF-negative cells do not express GFP/Cherry but demonstrated basal HGF levels. (C) HGF expression was monitored in paMSC-IGF-1-GFP populations (positive and negative). Positive populations were negative for HGF expression but the negative fraction showed basal HGF levels. (D) IGF-1 expression was monitored in paMSC-HGF-Cherry (positive and negative); both were positive for IGF-1 expression showing basal levels for this growth factor. (+) corresponds to primary and secondary antibodies ICC; (–) corresponds to ICC only with secondary antibodies. Secondary antibodies were Alexa-conjugated as in Additional file 4 (Figure S2). Scale bars = 20 μm. (TIF 242 kb) [file 13287_2016_350_MOESM5_ESM.tif]

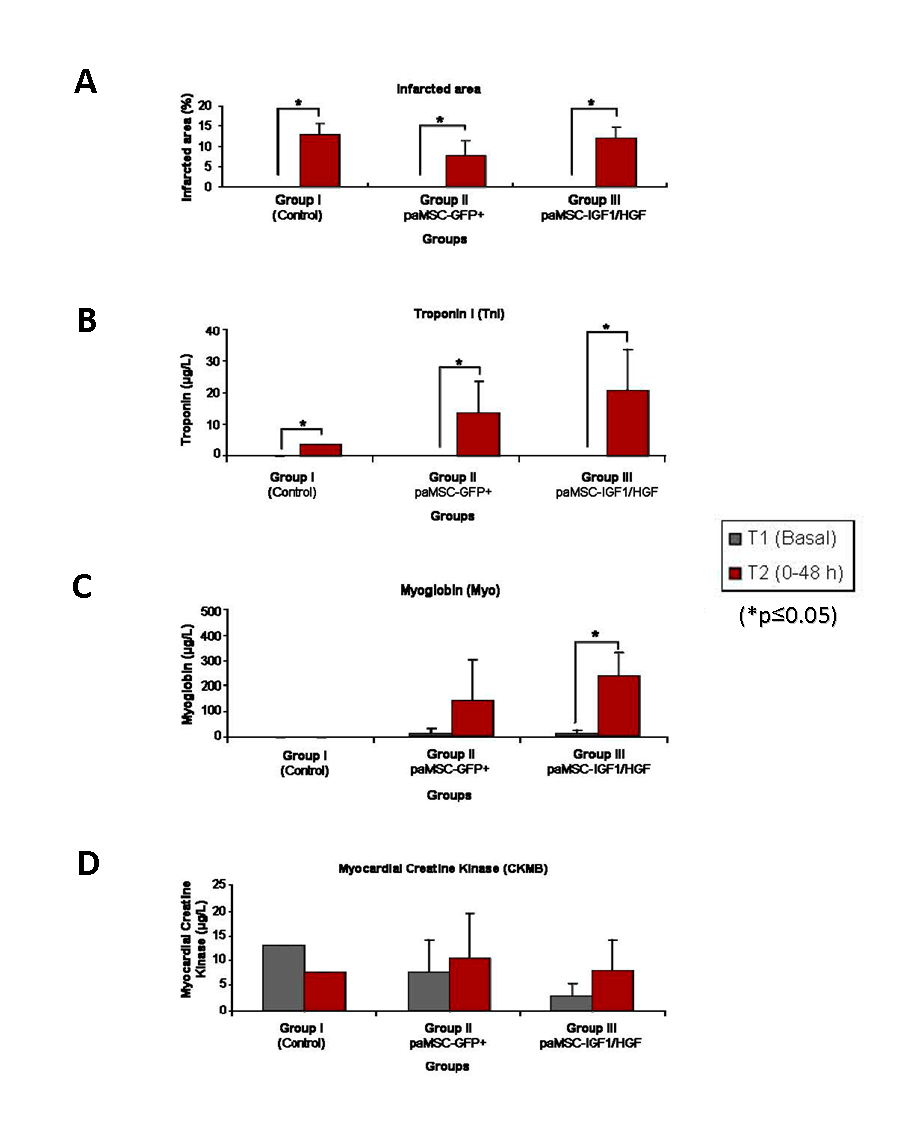

Supplement: Additional file 6: Figure S4. — Evaluation of infarct area and cardiac enzymes, comparing T1 vs T2. (A) Infarct area estimation, T1 vs T2. Analysis of (B) troponin I (TnI), (C) myoglobin (MYO) and (D) myocardial creatine kinase (CKMB) of all groups confirmed porcine AMI; (*p ≤0.05). (TIF 244 kb) [file 13287_2016_350_MOESM6_ESM.tif]

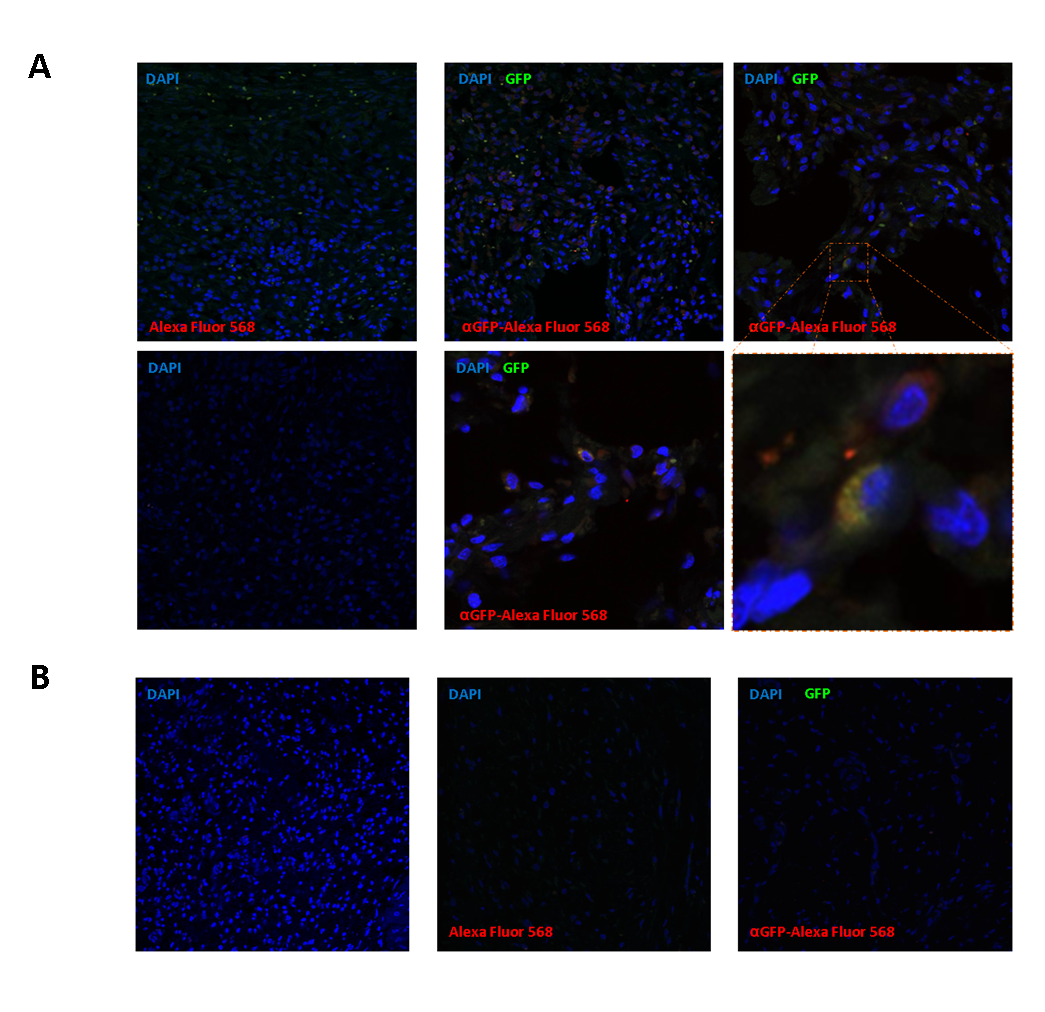

Supplement: Additional file 7: Figure S5. — (A) Immunohistocytochemistry analysis in control animals (transplanted but not infarcted). Animal transplanted with 30 × 106 paMSC + GFP + SPIO cells. Left panels show negative controls, incubation with Alexa 568-secondary antibody (top) and DAPI staining (bottom). Center and right images show isolated GFP-positive cells (anti-GFP-A568) in the Heart 2 section. (B) Immunohistocytochemistry analysis of control animals (no treatment). Left and center images show negative controls (DAPI staining and incubation with Alexa 568-secondary antibody). Right image shows negative results for GFP immunofluorescence. (TIF 1493 kb) [file 13287_2016_350_MOESM7_ESM.tif]

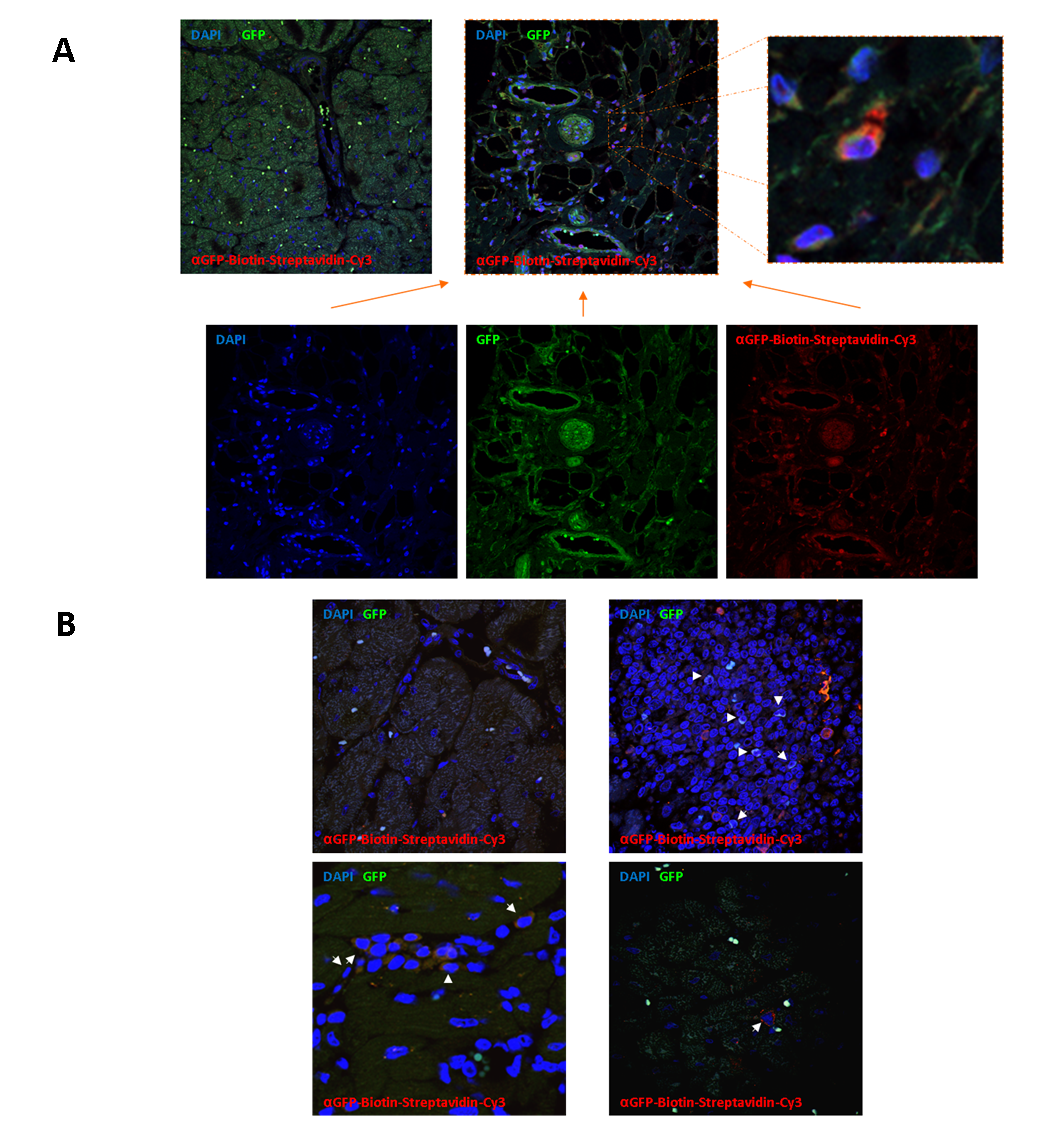

Supplement: Additional file 8: Figure S6. — Anti-GFP immunohistocytochemistry. (A) GFP-positive paMSC in a representative animal of group III (paMSC-IGF- 1-GFP/paMSC-HGF-Cherry). Bottom panel show serial cardiac sections (5 μm) counterstained with DAPI (left), imaged for GFP fluorescence (center) or anti-GFP-stained, and revealed with Cy3-streptavidin (right). Top central image corresponds to the merge of the three images shown in the bottom panel. Top left, negative control (group I) to determine tissue autofluorescence, stained with DAPI and anti-GFP- biotin and revealed with Cy3-streptavidin. Top right, an expanded view of the indicated area in the left image. (B) Sections from Heart 4 samples from animals representative of each group were stained with anti-GFP-biotin and revealed with Cy3-streptavidin, and counterstained with DAPI. Left top, control (group I); right top, transplanted uninfarcted animal; left bottom, group II (paMSC-GFP+); and right bottom, group III (paMSC-IGF-1-GFP/paMSC-HGF-Cherry) animals. (TIF 1592 kb) [file 13287_2016_350_MOESM8_ESM.tif]

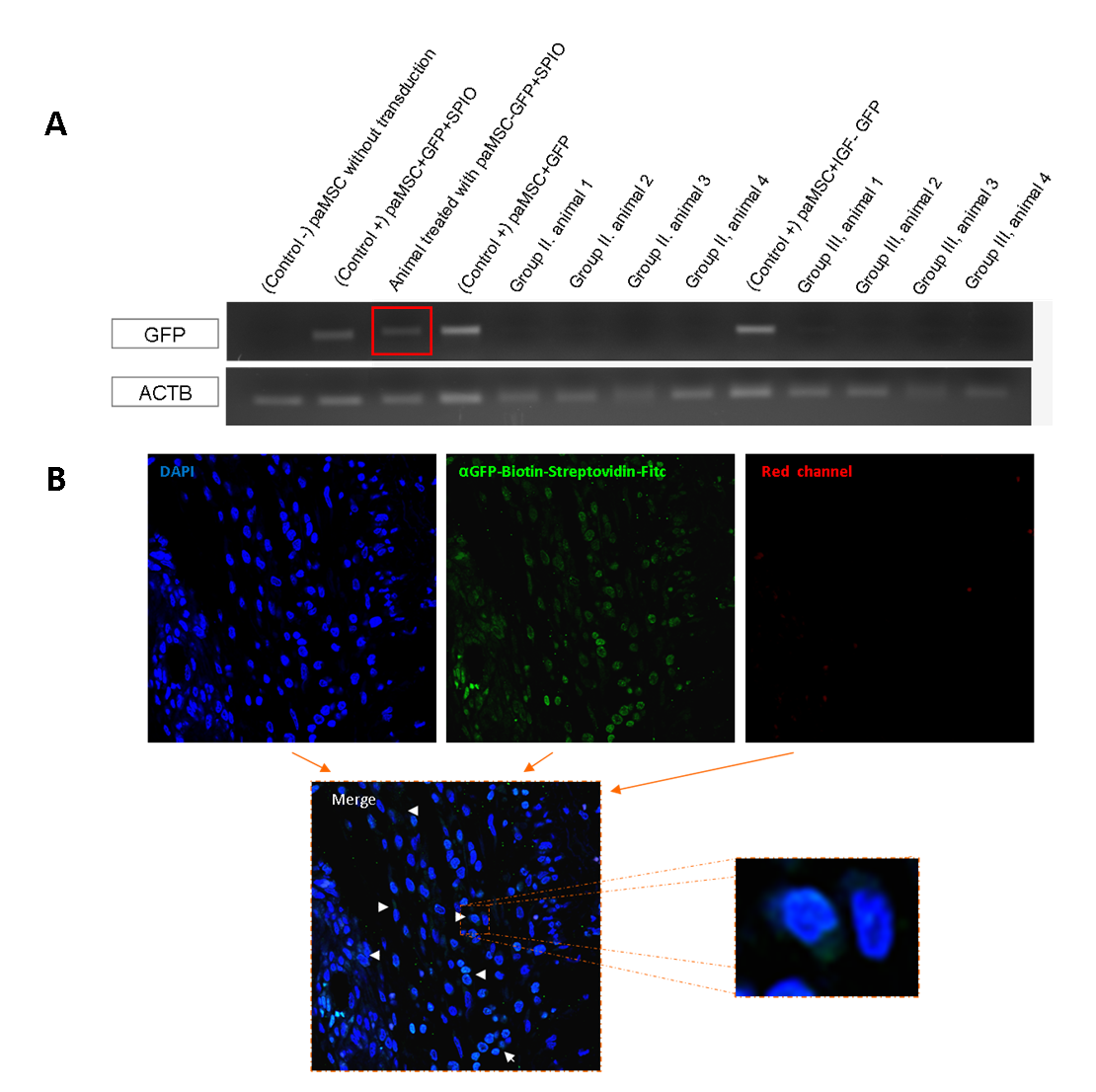

Supplement: Additional file 9: Figure S7. — (A) Molecular analysis of cardiac tissue. At 1 month post-transplant, GFP-positive cells were monitored in cardiac tissue (Heart 2) by genomic detection of GFP sequences (see Methods); ~100 bp diagnostic fragment. ACTB (135-bp fragment) was used for normalization. (B) Immunohistocytochemistry of anti-GFP with amplified FITC. Representative sample (Heart 4) sections from an animal transplanted but not infarcted were stained with anti-GFP-biotin and revealed with FITC-streptavidin, and counterstained with DAPI. (TIF 664 kb) [file 13287_2016_350_MOESM9_ESM.tif]

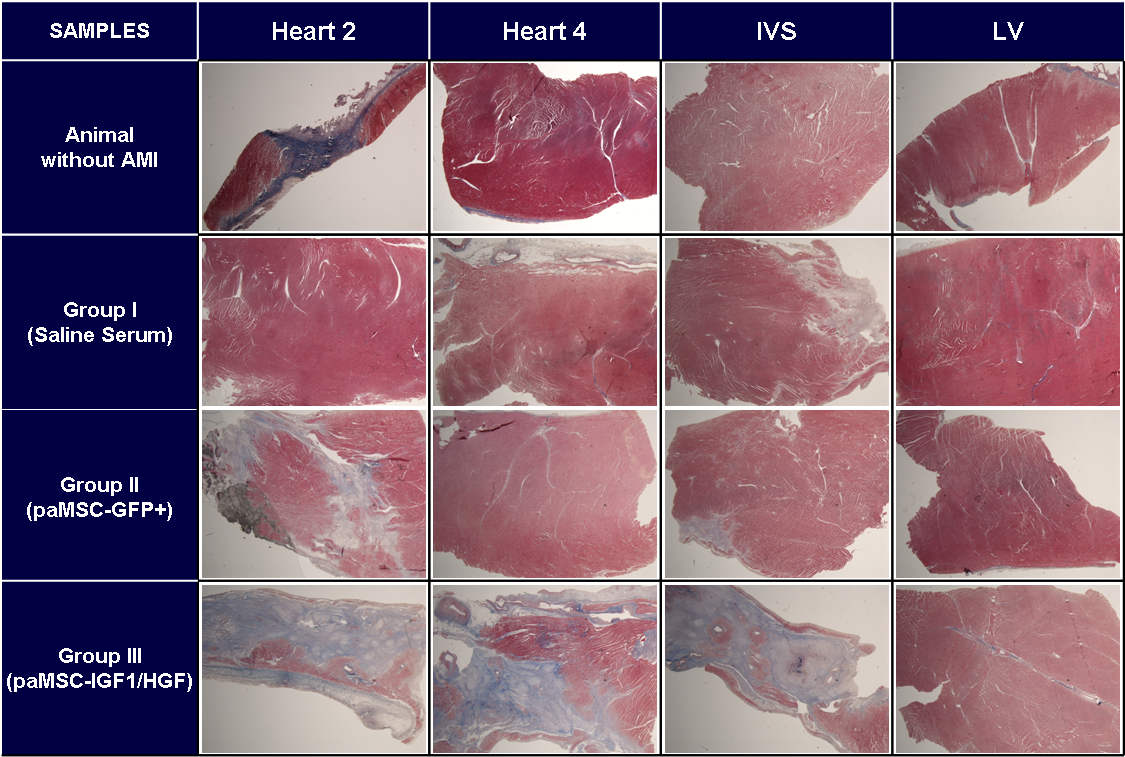

Supplement: Additional file 10: Figure S8. — Histomorphological study of cardiac tissue by Masson trichrome staining. Representative samples from each group, showing fibrotic areas (light blue) resulting from AMI (IVS inter-ventricular septum; LV left ventricle). (TIF 2164 kb) [file 13287_2016_350_MOESM10_ESM.tif]
